# Estimating the Cost-Effectiveness of HIV Prevention Programmes in Vietnam, 2006-2010: A Modelling Study

**DOI:** 10.1371/journal.pone.0133171

**Published:** 2015-07-21

**Authors:** Quang Duy Pham, David P. Wilson, Cliff C. Kerr, Andrew J. Shattock, Hoa Mai Do, Anh Thuy Duong, Long Thanh Nguyen, Lei Zhang

**Affiliations:** 1 Kirby Institute, University of New South Wales, Sydney, New South Wales, Australia; 2 Department for Disease Control and Prevention, Pasteur Institute, Ho Chi Minh City, Vietnam; 3 Department of Health System, Hanoi School of Public Health, Hanoi, Vietnam; 4 Department of Planning and Finance, Vietnam Administration of HIV/AIDS Control, Hanoi, Vietnam; 5 Ministry of Heath, Hanoi, Vietnam; 6 School of Public Health and Preventive Medicine, Faculty of Medicine, Nursing and Health Sciences, Monash University, Melbourne, Victoria, Australia; 7 Melbourne Sexual Health Centre, Alfred Health, Melbourne, Victoria, Australia; University of Washington, UNITED STATES

## Abstract

**Introduction:**

Vietnam has been largely reliant on international support in its HIV response. Over 2006-2010, a total of US$480 million was invested in its HIV programmes, more than 70% of which came from international sources. This study investigates the potential epidemiological impacts of these programmes and their cost-effectiveness.

**Methods:**

We conducted a data synthesis of HIV programming, spending, epidemiological, and clinical outcomes. Counterfactual scenarios were defined based on assumed programme coverage and behaviours had the programmes not been implemented. An epidemiological model, calibrated to reflect the actual epidemiological trends, was used to estimate plausible ranges of programme impacts. The model was then used to estimate the costs per averted infection, death, and disability adjusted life-year (DALY).

**Results:**

Based on observed prevalence reductions amongst most population groups, and plausible counterfactuals, modelling suggested that antiretroviral therapy (ART) and prevention programmes over 2006-2010 have averted an estimated 50,600 [95% uncertainty bound: 36,300–68,900] new infections and 42,600 [36,100–54,100] deaths, resulting in 401,600 [312,200–496,300] fewer DALYs across all population groups. HIV programmes in Vietnam have cost an estimated US$1,972 [1,447–2,747], US$2,344 [1,843–2,765], and US$248 [201–319] for each averted infection, death, and DALY, respectively.

**Conclusions:**

Our evaluation suggests that HIV programmes in Vietnam have most likely had benefits that are cost-effective. ART and direct HIV prevention were the most cost-effective interventions in reducing HIV disease burden.

## Introduction

Over the past 25 years, much has changed in Vietnam’s attempts to mitigate both the population and personal burden of HIV/AIDS. There was an absence of effective targeted prevention programmes across the country in the early response [1]. As a result, Vietnam was unable to curb the spread of HIV in the 1990s [2]. Later, Vietnam adopted community peer-based prevention interventions, with an ambitious target to alter the course of the increasing epidemic in many parts of the country [3, 4]. In particular, condom promotion programmes targeting female sex workers (FSWs) and their clients, and needle syringe exchange programmes (NSPs) targeting people who inject drugs (PWID), were rolled-out nationwide from 2002 and 2003, respectively. Since 2005, the national antiretroviral therapy (ART) programme has been rapidly scaled up according to World Health Organization (WHO) guidelines [5].

Like other low- and middle-income countries, international donors contribute the majority of funding for HIV programmes in Vietnam. Over 2008–2010, a total of US$363 million was invested in the HIV response [6]. About US$172 million was from the United States President's Emergency Plan for AIDS Relief (PEPFAR); US$15 million from the Global Fund, primarily supporting HIV care and treatment, and US$30 million from the United Kingdom Department for International Development (DFID) and the World Bank (WB) to support harm reduction interventions. In all, approximately 27.5% was from domestic resources and private sector, which primarily focus on mass communication, sexual health promotion programmes for youths, and surveillance. To date, several studies with limited scale have been conducted to examine the impacts of these programmes [7–10]. Although they provided important insights into some HIV programmes, their evaluations were of isolated programmes (e.g., methadone maintenance therapy (MMT) or ART only), without considering interactions with other interventions. At present, no national evidence is available to demonstrate both population health benefits of the entire response and value for the substantial investment in the HIV response in the Vietnamese context.

The objectives of this study were to investigate the possible population impacts and cost-effectiveness of the HIV programmes implemented in Vietnam over the period 2006–2010 using a retrospective modelling approach. We used a population-based model of HIV transmission, informed by the latest epidemiological and programming data at a national scale, to compare the differences in epidemiological outcomes between the status quo and assumed counterfactual scenarios representing what is assumed to have occurred programmatically and behaviourally in the absence of the programmes. This is then used to conduct to a cost-effectiveness analysis.

## Methods

### Data collation

We synthesised HIV epidemiological, programming, and costing data in Vietnam from two main sources. In Vietnam, the transmission patterns of HIV epidemics have been monitored annually by the national sentinel surveillance system [2, 3] and the periodic behavioural surveillance surveys [11] or integrated biological and behavioural surveillance [12, 13]. In addition to these national surveys, regional studies with smaller sample sizes were published in international peer-reviewed journals or as internal reports in in-country health organizations. These data were primarily collected through our ongoing collaboration with the Department for HIV Monitoring and Evaluation of the Vietnam Administration of HIV Control (VAAC). VAAC has provided data on population size estimates, HIV prevalence trends across population groups, and the number of HIV diagnoses and people living with HIV (PLHIV) on ART in the entire country over time. For independent studies in parallel with the sentinel surveillance, a systematic review of published English literature between 1990 and 2011 was conducted to collate data on HIV disease burden, HIV-related risk behaviours, and access to health promotion programmes in key affected populations (KAPs); further details are provided in S1 File.

Data on HIV spending in Vietnam during 2006–2010 were obtained through ongoing stakeholder collaborations with the authors. Comprehensive report in HIV financing—the National AIDS Spending Assessment [6]–was available for the period 2008–2010, but not earlier. To fill this data gap, we extracted relevant spending data from major national-level financial reports collected by our local data team and via comprehensive online searches for the period 2006–2007. The spending breakdown of total budgets to donors, programme areas and supporting costs was done by using proportional allocations available for the period 2008–2010 (Table B in S1 File). A prior study estimated that the costs of voluntary HIV counselling and testing (VCT) services were US$7.2 per client [14]. The healthcare costs for untreated HIV-positive people were within the range of US$105–178 per person-year, depending on levels of CD4^+^ T-cell counts; the annual costs for people receiving first-line and second-line ART were estimated to be US$344 and US$1,533, respectively (personal communication with Department of Planning and Finance, VAAC) [15].

### Model

We used the Optima model for the quantitative evaluation [16]. Details of this model are provided in S1 File. Briefly, Optima is a compartmental mathematical model of HIV that divided the overall 15–49 year old population in Vietnam into seven distinct risk populations as follows: males/females in the general population, direct/indirect FSWs, clients of sex workers, men who have sex with men (MSM), and PWID. Through a set of ordinary differential equations, the model tracks HIV transmission and the number of HIV-positive people and their rates of disease progression via four different CD4^+^ T-cell count categories (>500, 351–500, 200–350, and <200 cells/μL). The key model variable is the force-of-infection of HIV (λ), which is the probability with which uninfected individuals in each population become infected. It is expressed as λ = 1 −(1 − β)^n^, where β represents the transmission probability of HIV in each risk (sexual or injecting) event and *n* is the effective number of the events [17]. The model follows infected individuals through various stages of HIV infection and enables the calculation of disease burden in the populations at each of these stages. In this model, the Markov chain Monte Carlo algorithm was used to sample 1,000 sets of model parameters and then calibrate the model estimates to the observed epidemiological data [16]. A total of 40 calibrations with the best goodness-of-fit to the data were selected for computing summary statistics including median and 95% uncertainty bound. Further details are provided in S1 File.

### Effectiveness and cost-effectiveness analysis

Five programmes were selected for this evaluation including: NSPs and MMT for PWID, condom promotion for FSWs/clients and for MSM, and ART. Efficacy of these interventions in reducing HIV transmission in the target populations (e.g. 95% (85–99%) for consistent and correct condom use [18] and 96% (90–98%) for people who are virally suppressed while on ART [19, 20]) were included in the Optima model. The evaluation was based on defined counterfactuals, that is, assumptions about what would have occurred had the programmes not been implemented at a national level. For the counterfactuals, we assumed that key behavioural or coverage indicators associated with each programme (Table G in S1 File) would remain constant at the level prior to programme commencement. This approach differed from previous cost-effectiveness analyses of HIV programmes in Vietnam [8–10], which were largely dependent on the probability of behavioural change associated with a specific intervention for a single population (e.g., MMT for PWID). Our model, calibrated to reflect what actually occurred across population groups across the entire country, was used to simulate the epidemic according to these changed parameter assumptions. The model outputs were then compared between these two scenarios in terms of the estimated number of new infections, deaths, and DALYs for each programme and across all programmes. Absolute DALYs with the model for all scenarios were quantified by using available disability weights (Table F in S1 File) [21, 22]. We additionally assessed programme cost-effectiveness by estimating the costs required for averting one infection, HIV-related death, or DALY. We used an annual discount rate of 3%.

## Results

### Overview of HIV expenditure during 2006–2010

**Fig 1:**
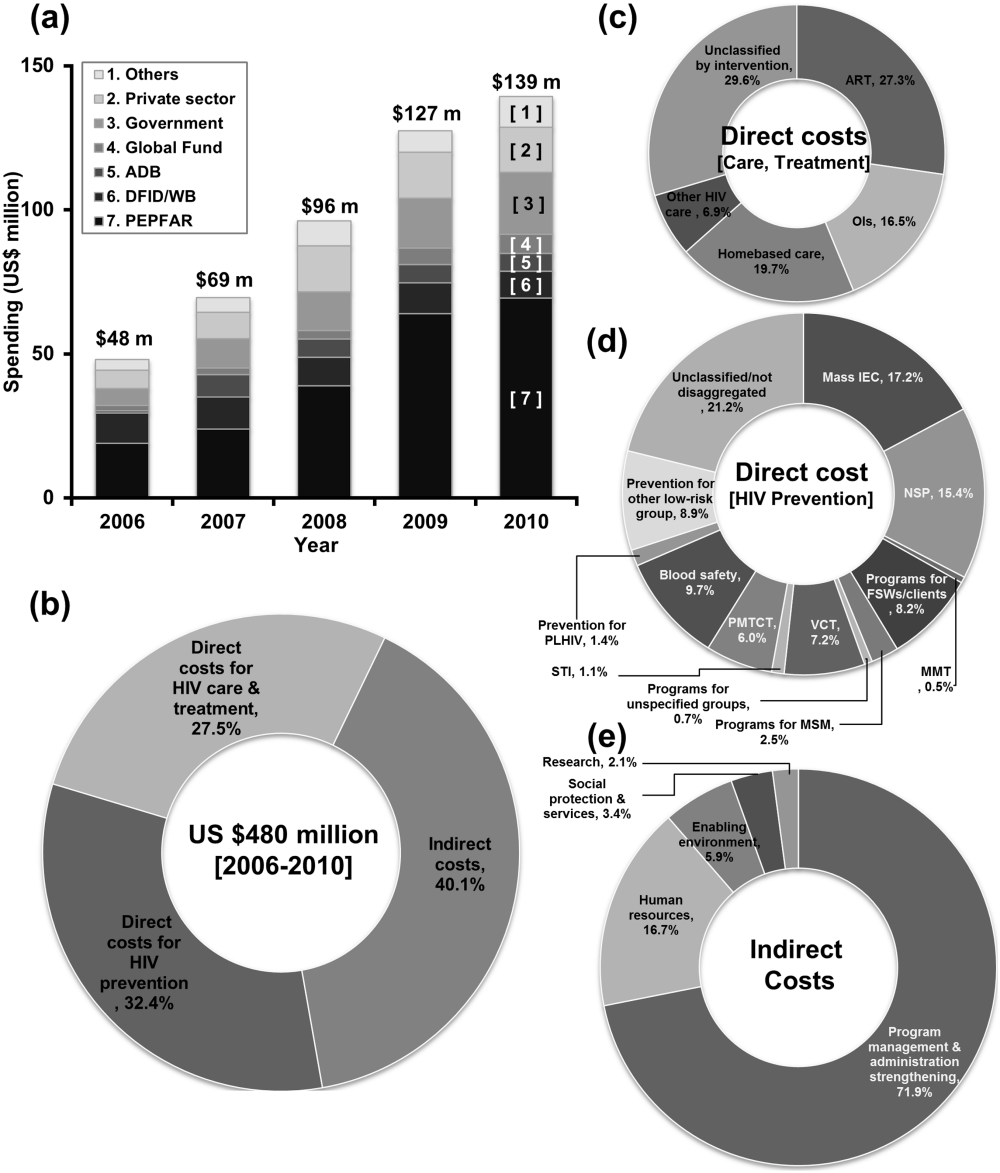
Budget allocation by donors and HIV programmatic areas, 2006–2010. (a) Overall HIV spending by donors. (b) Total funding by programmatic areas. (c) Direct cost for HIV care and treatment. (d) Direct cost for HIV preventions. (e) Indirect cost. ADB, Asian Development Bank; ART, antiretroviral therapy; DFID, the United Kingdom Department for International Development; FSW, female sex workers; IEC, information, education, and communication; MMT, methadone maintenance therapy; MSM, men who have sex with men; NSP, needle syringe exchange program; OIs, opportunistic infections; PEPFAR, the United States President's Emergency Plan for AIDS Relief; PLHIV, people living with HIV; PMTCT, preventing mother to child transmission of HIV; STI, sexually transmitted infections; VCT, voluntary HIV counselling and testing; WB, the World Bank.

Overall, about US$480.3 million was spent on the HIV programmes in Vietnam during 2006–2010 with annual spending nearly tripling from US$48.0 million in 2006 to US$139.2 million in 2010 (Fig 1a). The domestic contribution to the total HIV spending was at a low level and international sources accounted for most of funding (72.7%). PEPFAR was the largest source of support for HIV/AIDS programmes in Vietnam (44.8%) and was followed by DFID/WB (10.8%). From 2006 through 2010, expenditures for prevention programmes totalled US$155.8 million, with a substantial amount, of US$23.9 million (15.4%), spent on NSPs, followed by US$12.8 million (8.2%) for condom promotion programmes for FSWs and their clients (Fig 1d). Over the same period, US$132.1 million was directly allocated to HIV care and treatment programmes, with US$36.0 million (27.3%) directly spent on ART (Fig 1c). The amount of funding spent on FSW/clients was only one-half of the funding allocated to PWID despite a far greater numbers of new infections. Only US$3.8 million (2.5%) of the total funding for HIV prevention programmes was allocated to MSM who contributed 27.2% of all new infections during 2006–2010 (Figs 1d and 3a).

### Projected plausible epidemiological impacts of HIV programmes

**Fig 2:**
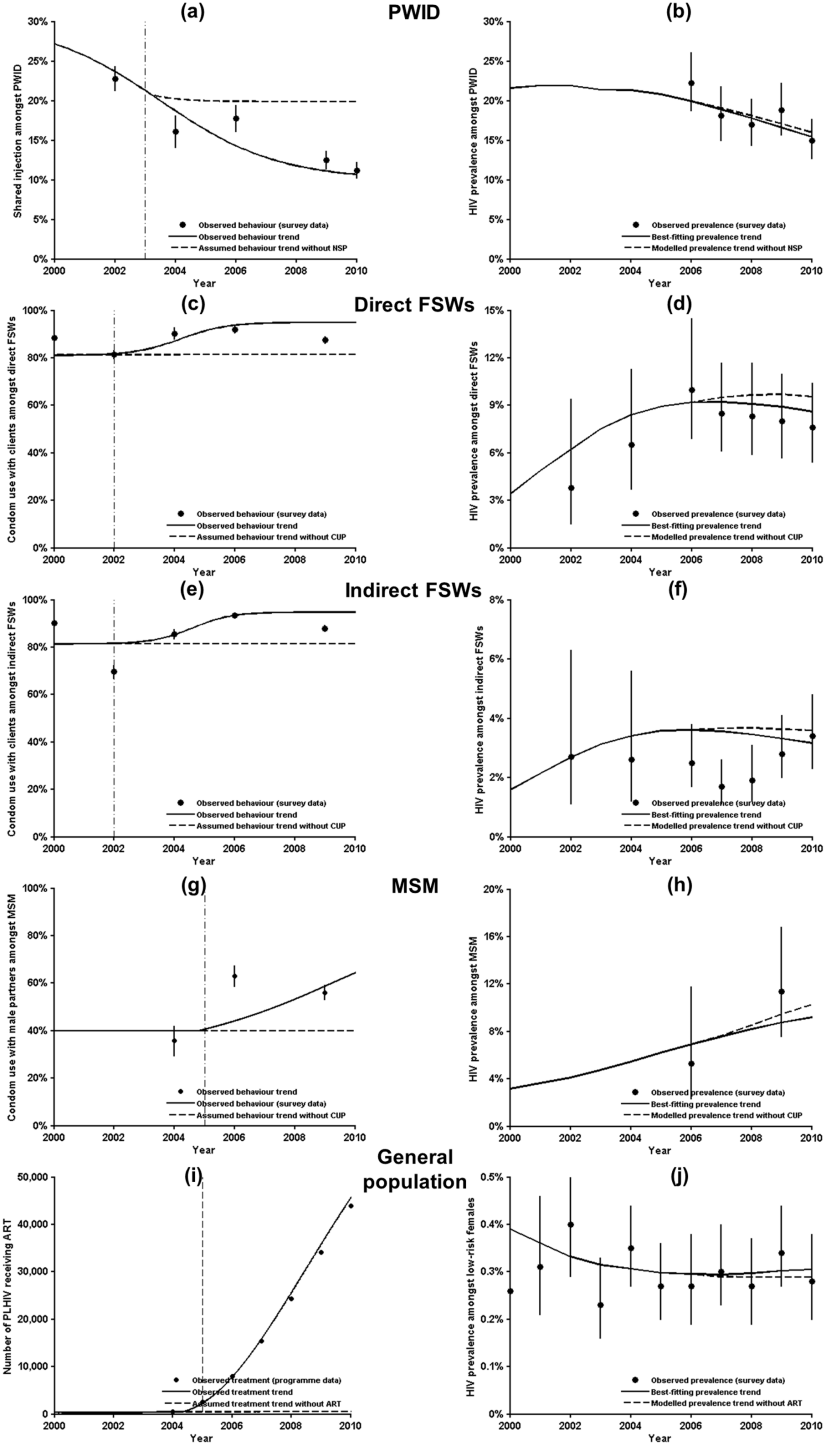
Observed HIV prevalence trends amongst key affected populations and low-risk females under the status quo versus projected prevalence trends in the absence of targeted programmes. Risk behaviours or interventions (left panels) and HIV prevalences (right panels) for each population group. HIV prevalence amongst pregnant women (as the low-risk female population) was assumed to be representative for the prevalence amongst the general population. Curves presented in the five figures in the left column were obtained from a generalised 4-parameter logistic function, whereas curves in the five right-hand figures were model trajectories. Solid curves represent median of 40 best-fitting trends under the status quo. Dashed curves represent assumed behaviour or modelled prevalence trends in the absence of programmes (i.e. the counterfactual). Black dots with solid vertical lines represent observed survey data with 95% confidence intervals. The vertical dash line indicates the initial year of programme implementation.

Behaviour trends indicated lower risk behaviours over time amongst PWID and different groups of FSWs (Fig 2a, 2c and 2e). Condom use by MSM was increasing, but remained at low levels over the study period (Fig 2g). Despite these changes, our model trajectories of HIV prevalence were within confidence intervals of observed data. However, prevalence trends decreased amongst PWID and FSWs associated with the reduction in risk behaviour. According to our assumed counterfactual scenarios of coverage and risk levels without the interventions, the prevention programmes reduced prevalence and brought a decline in prevalence amongst KAPs, particularly PWID and FSWs (Fig 2b, 2d and 2f), but not MSM (Fig 2h). A low but gradually increasing percentage of condom use amongst MSM under the status quo, given the relatively limited investment in HIV programmes targeting MSM, would contribute to the slowing down of HIV transmission in MSM but was impossible to reserve its rising trend. Of note, implementation of the ART programme had led to a slightly higher HIV prevalence in general population, as compared to the counterfactual scenario in which ART was absent (0.30% versus 0.29% in 2010, respectively, Fig 2j), due to its large population benefits in reducing the numbers of HIV-related deaths and incident infections (41,616 [95% uncertainty bound: 35,406–53,515] and 14,249 [10,426–21,23], respectively, Table 1).

**Table 1:** Estimated effectiveness and cost-effectiveness of past HIV/AIDS programmes in Vietnam, 2006–2010.

	Programme cost	Estimated infections averted (95% UB)	Estimated deaths averted (95% UB)	Estimated DALYs averted (95% UB)	Programme cost per infection averted (95% UB)	Programme cost per death averted (95% UB)	Programme cost per DALY averted (95% UB)
Needle and syringe programme	$23,935,822	5,212 (2,761, 7,522)	338 (160, 517)	16,306 (8,533, 24,253)	$4,592 (3,182, 8,669)	$70,816 (46,298, 149,599)	$1,493 (987, 2,805)
Methadone maintenance therapy programme	$853,296	Nil	Nil	Nil	Nil	Nil	Nil
Condom promotion programme for FSWs	$12,769,281	12,035 (8,467, 20,240)	1,022 (748, 1,421)	42,231 (30,817, 64,150)	$1,061 (631, 1,508)	$12,494 (8,986, 17,071)	$302 (199, 414)
Condom promotion programme for MSM	$3,838,991	10,652 (3,080, 13,797)	678 (250, 810)	37,190 (10,874, 45,509)	$360 (278, 1,246)	$5,662 (4,739, 15,356)	$103 (84, 353)
Antiretroviral therapy programme (including PMTCT)	$45,392,265	14,249 (10,426, 21,023)	41,616 (35,406, 53,515)	276,401 (221,365, 349,243)	$3,186 (2,159, 4,354)	$1,091 (848, 1,282)	$164 (130, 205)
All programmes^a^	$99,747,684	50,570 (36,314, 68,921)	42,557 (36,070, 54,129)	401,550 (312,207, 496,278)	$1,972 (1,447, 2,747)	$2,344 (1,843, 2,765)	$248 (201, 319)

DALY, disability adjusted life-year; FSW, female sex workers; MSM, men who have sex with men; PMTCT, prevention of mother-to-child transmission of HIV; UB, uncertainty bound of model simulations.

^a^This included expenditure on NSP, MMT programme, condom use promotion programmes for FSWs and MSM, sexually transmitted infections programme, voluntary HIV counselling and testing programme, and ART.

**Fig 3:**
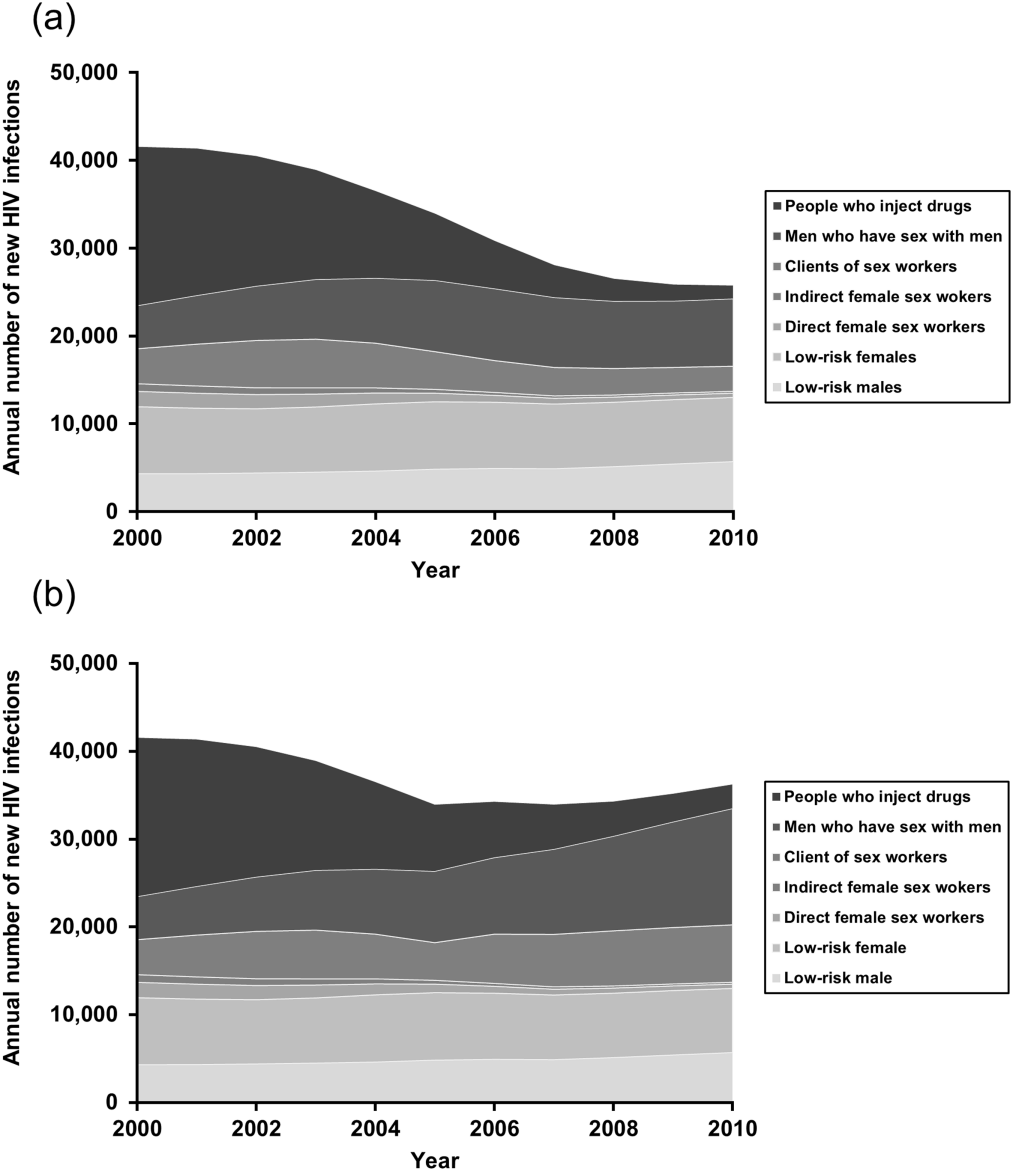
Projected median annual number of new HIV infections in Vietnam by population groups, 2000–2010. (a) Status quo. (b) Counterfactual scenario (without programmes).

Our modelling indicates that Vietnam’s HIV investments between 2006 and 2010 have averted a total of 50,570 [36,314–68,921] infections and 42,557 [36,070–54,129] deaths, corresponding to a 33.5% and 35.2% reduction of population numbers of new infections and deaths, respectively (Table 1). Furthermore, we estimated 27,180 [20,770–53,390] new infections occurring in Vietnam in 2010, showing a 35.4% reduction from 42,102 [36,811–53,826] cases in 2001 (Fig 3a). Whereas there has been a considerable reduction in new infections amongst both PWID and FSWs/clients over 2006–2010, approximately 8,000 MSM were newly infected with HIV every year since 2005. Among the five targeted programmes, the most effective intervention in reducing new infections was estimated to be the ART programme (14,249 [10,426–21,23], 9.5% of all new infections). It was followed by condom promotion programmes for FSWs/clients (12,035 [8,467–20,240], 7.8%), MSM (10,652 [3,080–13,797], 7.1%), and NSPs (5,212 [2,761–7,522], 3.5%). The prevention programmes were estimated to have led to a total of 401,550 [312,207–496,278] fewer DALYs over 2006–2010 (Table 1).

### Projected cost-effectiveness of HIV programmes

With a total amount of US$480.3 million spent during 2006–2010, each infection and HIV-related death averted cost an estimated US$9,498 [6,969–13,227] and US$11,287 [8,874–13,317], respectively. Yet these costs were about five times lower when just direct investment on HIV prevention and ART programme costs were considered (US$1,972 [1,447–2,747] and US$2,344 [1,843–2,765], respectively). Each DALY averted was estimated to cost approximately US$1,196 [968–1,538] when the entire HIV investment was included and only US$248 [201–319] when only direct programme costs were included (Table 1).

## Discussion

Vietnam has had a declining HIV epidemic trend between 2006 and 2010 [23]. ART, NSPs and condom promotion programmes for FSW/clients and MSM are likely to have been largely responsible for bringing about the decline. In this study, we found that there is not convincing evidence of population impact. Nevertheless, our model-based estimates suggest that HIV programmes have averted approximately 50,600 infections, 42,600 deaths, and 401,600 DALYs over 2006–2010, corresponding to costs of US$1,972, US$2,344, and US$248 per infection, death, and DALY averted, respectively. According to most willingness-to-pay thresholds [24], these ratios are likely to be deemed highly cost-effective.

Condom promotion programmes for FSWs/clients have demonstrated plausible moderate population impacts as about a quarter of the averted new infections during 2006–2010 were attributed to this programme. Despite being cost-effective, its cost-effectiveness ratios are much higher than countries with similar national FSW programmes (in India: US$104 and US$11 for each infection and DALY averted) [25]. The difference may be partly explained by the shorter duration of our evaluation (5-year versus 20-year time horizon). This ratio may become similar to those studies for other settings [26] if the currently large amount of indirect costs associated with programme administration and management could be reduced.

NSPs have had a major and noticeable impact on the HIV epidemic in Vietnam, which adds to a growing body of global evidence of the effectiveness of the NSPs in reducing HIV transmission amongst PWID [27]. The finding is strongly supported by the low level of receptive sharing of syringes recently reported amongst Vietnamese PWID (13% in the past month in 2009) [13], which is already comparable to those in high-income settings (e.g., Australia, 16% in 2009 [28]; Canada, 15% in 2006 [29]). Ongoing funding for NSPs is essential to curb the HIV epidemic in Vietnam, despite the estimated costs per infection averted by Vietnam’s NSPs found to be considerably higher than that of local NSPs in other low- and middle-income countries with a rising burden of HIV amongst PWID such as Odessa, Ukraine (US$97) [30], Dhaka, Bangladesh (US$110) [31], Yunnan, China (US$138–198) [32], and Svetlogorsk, Belarus (US$359) [33]. The higher cost per infection of Vietnamese NSPs may be explained by the large-scale implementation of the programmes that is often associated with higher cost requirements and an apparent decline in new HIV infections amongst Vietnamese PWID over the study period. Unlike NSPs, which have been long implemented in areas of Vietnam, the low-level coverage of MMT since its initial roll out in 2008, estimated to be 5.6% by 2012 [34], has limited our attempts to evaluate its population health effects. Future studies are required to better define the effects of this programme for PWID in Vietnam. Despite this, continued scale-up of MMT is likely to improve the overall impact and cost-effectiveness of the HIV response in Vietnam [35]. MMT also leads to other social and public health gains [36, 37].

Our projection of a rapidly expanding HIV epidemic amongst MSM suggests a shift in the modes of HIV transmission in Vietnam. It is possible that the limited investment in HIV prevention programmes targeting MSM over the past decade in this country has substantially fuelled homosexual HIV transmission. Shifting prevention resources to this population, which is known to be at a much higher risk of HIV infection globally [38], is urgently needed to curb the epidemic. Specifically, promotion of condom use by MSM is a priority strategy to prevent the sexual transmission of HIV to their serodiscordant same-sex partners, as well as to limit the spread of HIV into the wider community through unprotected sexual activities with their female partners [13]. Overlapping sexual and injecting behaviours are also common amongst Vietnamese MSM [39], which makes it important to coordinate condom and harm reduction programmes for drug-using MSM to minimise the cross-transmission of HIV.

Cohen and colleagues [19] showed substantial prevention benefits of providing early ART in reducing heterosexual transmission of HIV in serodiscordant couples, from which the treatment as prevention (TasP) strategy has been recommended for many settings. Despite the potentially large preventive benefits of Vietnam’s ART programmes and the low cost per infection of the public-health ART approach used in Vietnamese ART programme [5, 15, 40], as observed in this study, it is important to emphasize that there are several key barriers for a successful roll-out of TasP in Vietnam. The first is the constrained government budget for HIV interventions, the weakness of the government-funded HIV healthcare system, and a shortage of health professionals. These difficulties make the TasP strategy difficult to roll out and scale up at the national level in the immediate future. Alternatively, a narrower scope of early and immediate initiation of ART regardless of CD4^+^ T-cell count level for specific HIV-positive subgroups, as per recent recommendations of the WHO [41, 42], may be a feasible option for Vietnam. Low access to HIV treatment amongst Vietnamese PLHIV is another hurdle. Despite a significant effort since 2005 to rapidly scale up HIV testing and ART for PLHIV, by 2012, amongst the 197,300 people diagnosed with HIV in Vietnam, fewer than half were linked to HIV care and only a third were on ART [43]. Thus a critical area of need in Vietnam is an improved continuum of HIV care and treatment through timely diagnosis, referral to HIV care, and early ART initiation for eligible PLHIV.

With a heavy reliance on international donors for the HIV response and a foreseeable reduction in funding in the near future, a scale-up of domestic revenue to maintain effective responses to the epidemic in Vietnam is required. The limited resources mean every dollar for HIV responses needs to be spent effectively. A recent study on population-level cost-effectiveness of the large community-based Avahan programme in south India strongly indicated that KAP-focused interventions are highly cost-effective [44]. Our findings recommend a shift of available resources away from the general populations and towards the KAPs in Vietnam, which is consistent with a recent systematic review of HIV funding landscape in other Asian settings [45]. NSPs and condom programmes amongst MSM and FSW/clients should be specifically prioritised.

Our study has several limitations. Empirical data on the actual effects of an intervention for KAPs on changing their high-risk behaviours and incidence rates between the intervention and control groups have been lacking in Vietnam. Therefore, we could not perform an evaluation of evidence-based interventions in this study with a well-defined control group (or counterfactual). Instead, this evaluation depends on assumptions of conditions remaining unchanged had the interventions not taken place. It is possible that risk behaviours may have decreased without investment in the specific interventions. Furthermore, behavioural and surveillance data were taken from a series of cross-sectional surveys amongst KAPs, which may not be completely comparable over time and thus contribute in part to wide uncertainty bounds of the findings. Several other model parameters used in our analysis also have large uncertainties. For example, the HPTN052 clinical trial demonstrated a 96% reduction in rates of sexual transmission amongst serodiscordant heterosexual couples in nine African countries [19], whereas no difference or a smaller reduction (26%) in HIV risks were found from other observational studies amongst Ugandan and Chinese serodiscordant couples [46, 47]. More importantly, preventive effects of ART on homosexual and injection sharing transmission of HIV are still unknown and assumed in this study to be identical to that of heterosexual transmission. In a national evaluation, Nguyen *et al*. [48] further showed a low CD4^+^ cell count among people initiating therapy for HIV, indicating a long lag time from HIV infection to HIV diagnosis and care among Vietnamese people living with HIV. This may suggest that the observed decline in diagnosis over the study period of 2006–2010 may be contributed in part by the effects of the HIV programmes implemented in the earlier period (i.e., 2000–2005). However, our study was hindered by incomplete costing data for the period 2000–2005, which has limited our further investigation of the relationship between HIV spending with key behavioural indicators. Spending data were not stratified by provincial/regional levels, so the cost-effectiveness of programmes in specific Vietnamese provinces/regions could not be assessed in this analysis. Thus, while we used the best available evidence to conduct this study, the extreme paucity of empirical data and the resultant reliance on informed assumptions renders it impossible to make definitive claims about the effectiveness of the programmes considered.

### Conclusions

Our model-based estimates suggest that HIV programmes in Vietnam have most likely been cost-effective in reducing the HIV disease burden; however, with counterfactual projections overlapping with intervals around observed data, the evidence for effectiveness is only moderate. With a highly concentrated epidemic in Vietnam, it is imperative to shift HIV resources from programmes for the general population towards programmes for the KAPs for greatest population benefits.

## Supporting Information

S1 File Description of the mathematical model and parameter values. Flow chart of article selection (**Figure A**). HIV prevalence among various populations in Vietnam, 2000–2011 (**Figure B**). Number of reported HIV diagnosis in Vietnam, 2000–2011 (**Figure C**). Number of PWID registered methadone maintenance therapy (**Figure D**). Number of people receiving ART in Vietnam, 2006–2010 (**Figure E**). Model schematic for HIV infection progression (**Figure F**). Calibrated input parameters by Optima (**Figure G**). Projected prevalence levels are shown by population group (**Figure H**). Summary of literature among key affected populations in Vietnam (**Table A**). Estimated HIV spending in Vietnam, 2006–2010 (**Table B**). Population interactions (**Table C**). Mathematical modelling inputs (**Table D**). Healthcare costs of HIV infected people in 2009 (**Table E**). Disability-weights for cost effectiveness calculations (**Table F**). Selected behaviours affected by HIV prevention programmes (**Table G**).
(DOCX)
