# Supplementary material for: Estimating the Cost-Effectiveness of HIV Prevention Programmes in Vietnam, 2006-2010: A Modelling Study
Source: PLoS One. 2015 Jul 21;10(7):e0133171. doi: 10.1371/journal.pone.0133171 (PMC4510535; doi:10.1371/journal.pone.0133171)
Supplement: S1 File — Flow chart of article selection (Figure A). HIV prevalence among various populations in Vietnam, 2000–2011 (Figure B). Number of reported HIV diagnosis in Vietnam, 2000–2011 (Figure C). Number of PWID registered methadone maintenance therapy (Figure D). Number of people receiving ART in Vietnam, 2006–2010 (Figure E). Model schematic for HIV infection progression (Figure F). Calibrated input parameters by Optima (Figure G). Projected prevalence levels are shown by population group (Figure H). Summary of literature among key affected populations in Vietnam (Table A). Estimated HIV spending in Vietnam, 2006–2010 (Table B). Population interactions (Table C). Mathematical modelling inputs (Table D). Healthcare costs of HIV infected people in 2009 (Table E). Disability-weights for cost effectiveness calculations (Table F). Selected behaviours affected by HIV prevention programmes (Table G). (DOCX) [file pone.0133171.s001.docx]

**S1 File. Description of the mathematical model and parameter values**

Quang D. Pham, David P. Wilson, Cliff C. Kerr, Andrew J. Shattock, Hoa M. Do, Anh T. Duong, Long T. Nguyen, Lei Zhang

Table of Contents

[1. Collection of Epidemiological and Behavioural Data in Vietnam 1](#_Toc418538388)

[2. Description of Mathematical Model 12](#_Toc418538389)

[3. Model Parameters 15](#_Toc418538390)

[4. Counterfactual Scenarios in the Absence of HIV Interventions 21](#_Toc418538391)

[5. Calibration of model to the HIV epidemic in Vietnam 22](#_Toc418538392)

1. Collection of Epidemiological and Behavioural Data in Vietnam

***HIV epidemiological, programming, and clinical data***

In Vietnam, the transmission pattern of HIV epidemics is monitored by the national sentinel surveillance system annually and the periodic integrated biological and behavioural surveillance (IBBS) and behavioural surveillance surveys (BSS). Moreover, regional studies with substantially smaller sample sizes are published in international peer-reviewed journals or as internal reports in in-country health organizations. For independent studies outside sentinel surveillance, a systematic review was conducted to collating relevant parameters from published English literatures during 1990-2011. We performed searches in May 2011 for on PubMed according to the following key word search strategies:

*Epidemiological and behavioural parameters among drug users*

- [‘HIV’ OR 'human immunodeficiency virus’ OR ‘STI’ OR ‘STIs’ OR ‘STD’ OR 'sexually transmitted infection' OR 'sexually transmitted disease'] AND [‘IDU’ OR ‘injecting drug user’ OR ‘injection drug user’ OR ‘drug user’ OR ‘heroin user’ OR ‘substance user’ OR ‘opioid use’ OR ‘methadone’ OR ‘methadone maintenance therapy’ OR ‘methadone maintenance treatment’ OR ‘MMT’ OR ‘injecting behaviour’ OR ‘injecting behaviour’ OR ‘sharing behaviour’ OR ‘sharing behaviour’ OR ‘sharing practice’ OR ‘injection practice’ OR ‘injecting practice’] AND [‘Vietnam’];

*Epidemiological and behavioural parameters among female sex workers*

- [‘HIV’ OR 'human immunodeficiency virus’ OR ‘STI’ OR ‘STIs’ OR ‘STD’ OR 'sexually transmitted infection' OR 'sexually transmitted disease'] AND ['FSW' OR 'female sex worker' OR 'sex work' OR 'sex worker' OR 'condom' OR 'condom use' OR 'condom practice' OR 'sexual behaviour' OR 'sexual behaviour' OR 'sexual partner' OR 'sexual partnership'] AND [‘Vietnam’];

*Epidemiological and behavioural parameters among men who have sex with men*

- [‘HIV’ OR 'human immunodeficiency virus’ OR ‘STI’ OR ‘STIs’ OR ‘STD’ OR 'sexually transmitted infection' OR 'sexually transmitted disease'] AND [‘MSM’ OR ‘men who have sex with men’ OR ‘gay’ OR ‘bisexual’ OR ‘homosexual’ OR ‘same sex’ OR ‘male sex worker’ OR ‘MSW’ OR ‘transgender’ OR ‘homosexual’ OR ‘homosexuality’] AND [‘Vietnam’];

*HIV intervention programs in Vietnam*

- [‘HIV prevention’ OR ‘HIV intervention’ OR ‘HIV program’] AND [‘Vietnam’].

In this review, a publication was included if it reported prevalence levels of HIV and/or sexually transmitted infections (STI), sexual and/or drug-using behaviours among key affected populations (KAPs including people who inject drugs (PWID), female sex workers (FSW), clients of FSWs, and men who have sex with men (MSM)) in Vietnam. Our review excluded systematic review and qualitative studies, studies prior to 1^st^ January 2000, or sample size less than 30 and publications from the identical data source.

**Fig A. Flow chart of article selection.**

Eligible articles identified (n=41)

Articles identified through searching PubMed database
(n=691)

Grey-literatures included
 (n = 9)

Title and abstracts screened (n=198)

Studies included in synthesis (n=50)

**Screening**

**Included**

**Eligibility**

**Identification**

Articles assessed for eligibility (n=53)

Data not belong to KAPs
(n=145)

Records excluded (n=12)

- Duplications (n=4)
- Prior 1^st^ January 2000 (n=2)
- Antiretroviral therapy (n=4)
- Cost-effectiveness of HIV programs (n=2)

For this study, HIV sentinel data was provided by the Vietnam Authority of HIV/AIDS Control for the period 2000-2010. Additional prevalence data was also collected through a systematic review of published peer-reviewed research articles published after 1^st^ January 2000 by searching the Medline database through PubMed. In brief, a total of 691 articles were identified through our search strategy and were screened but after applying strict exclusion criteria, 41 independent published studies were chosen to provide HIV prevalence or risk behavioural data among key populations over the period 2000-2010. Additional nine grey-reports were also collected by in-country consultants. A total of 50 literatures were included in this systematic review (Fig. A and Table A). These studies represent a total of 20,417 of PWID screened in 22 provinces, 22,301 FSWs screened in 17 provinces, and 3,805 MSM screened in six provinces, these numbers represent 9.4%, 34.3%, and 1.0% of population size estimates in the entire country, respectively.

Required data were extracted and entered into a database using *Microsoft Access*. Meta-analyses on HIV prevalence data were carried out using the Comprehensive Meta-Analysis software version 2.0 (Biostat, Englewood, New Jersey). We used random effect models to estimate the pooled HIV prevalence and its 95% confidence intervals. We used a similar method as a preceding report of evaluation of harm reduction programmes in Vietnam occurring during 2009 and 2010 to estimate the weight average of key behaviours (i.e. sharing rate, estimated number of partner per year, and percentage of condom use with different partnerships) in various groups [1]. In this analysis, we only included surveys which enrolled participants in several provinces (i.e. BSS 2000, Baseline in 2002 and endpoint survey of the project of “Community Action for Preventing HIV/AIDS”, and IBBS 2006 and 2009).

The trend of HIV prevalence between 2000 and 2012 was presented in seven high-risk (including PWID, MSM, street-based FSWs, entertainment-based FSWs, male STI patients) and low-risk populations (including male military recruits, and pregnant women) (Fig. B). A first-ever effort to determine HIV prevalence and risk behaviours in clients of FSWs through a well-designed cross-sectional survey in two tourist towns in Hai Phong in early 2007 showed a HIV prevalence of 4.5% (95% CI: 1.9 – 5.6%) [2], which is within the HIV prevalence range of 2.3 – 4.5% found among male STI patients in that province during 2006-2008. Therefore, in this study, we used the observed HIV prevalence among male STI patients as a proxy indicator of HIV prevalence among clients of FSWs. The trend of key available behavioural parameters is indicated among four sub-groups, including PWID, MSM, street-based FSWs, and entertainment-based FSWs (Table D).

Program and costing data at the national level was obtained through in-country consultants. These include the number of people diagnosed with HIV, number of PWID receiving methadone maintenance therapy (MMT), and number of people receiving antiretroviral therapy (ART) in Vietnam, by contacting with key central project management units and the Vietnam Authority of HIV/AIDS Control (Figs. C, D, and E). In the process of data collection, we also collated healthcare costs of HIV infected people and disability weight for cost effectiveness calculations (Tables E and F, respectively).

**Table A. Summary of literature among key affected populations in Vietnam.**

| **First author, published year (Ref)** | **Sample duration** | **Study province** | **HIV prevalence** | | |
| --- | --- | --- | --- | --- | --- |
|  |  |  | **n** | **N** | **Percent** |
| **People who inject drugs** |  |  |  |  |  |
| Hien et al., 2000* [3] | 04/1997 | HCMC | - | 630 | - |
| Hien et al., 2001 [4] | 04/1997 | HCMC | 76 | 300 | 25.3% |
| Hien et al., 2001 [4] | 04/1998 | HCMC | 126 | 296 | 42.6% |
| Nguyen et al., 2001 [5] | 03-05/1999 | Haiphong | 93 | 201 | 46.3%^[[1]](#footnote-1)^ |
| Nguyen et al., 2001 [5] | 03-05/1999 | Haiphong | 235 | 319 | 73.7% |
| Nguyen et al, 2001* [6] | 06-11/2000 | Cantho | - | 384 | - |
| Nguyen et al, 2001* [6] | 06-11/2000 | Danang | - | 297 | - |
| Nguyen et al, 2001* [6] | 06-11/2000 | HCMC | - | 420 | - |
| Nguyen et al, 2001* [6] | 06-11/2000 | Hanoi | - | 360 | - |
| Nguyen et al, 2001* [6] | 06-11/2000 | Haiphong | - | 326 |  |
| Thao et al., 2006^†^ [7] | 10/1999-03/2000 | HCMC | 50 | 400 | 8.0% |
| Nguyen et al., 2007 [8] | 04-06/2002 | Angiang | 48 | 358 | 13.4% |
| Nguyen et al., 2007 [8] | 04-06/2002 | Dongthap | 11 | 274 | 4.0% |
| Nguyen et al., 2007 [8] | 04-06/2002 | Kiengiang | 94 | 400 | 23.5% |
| Nguyen et al., 2007 [8] | 04-06/2002 | Laichau | 130 | 359 | 36.2% |
| Tran et al., 2006 [9] | 09-11/2002 | Longan | 80 | 248 | 32.3% |
| Des Jarlais et al., 2007 [10] | 06/2002 | Langson | 17 | 55 | 30.9% |
| Des Jarlais et al., 2007 [10] | 12/2002 | Langson | 16 | 64 | 25.0% |
| Hammett et al., 2006 [11] | 06/2002 | Langson | 157 | 342 | 45.9% |
| Hammett et al., 2006 [11] | 12/2002 | Langson | 156 | 340 | 45.9% |
| Bergenstrom et al, 2007 [12]; Quan et al., 2009 [13] | 08-09/2003 | Bacninh | 131 | 309 | 42.4% |
| Go et al., 2006* [14];  Schumacher et al, 2008* [15] | 08-09/2003 | Bacninh | - | 393 | - |
| Des Jarlais et al., 2007 [10] | 06/2003 | Langson | 23 | 104 | 22.1% |
| Des Jarlais et al., 2007 [10] | 12/2003 | Langson | 14 | 74 | 18.9% |
| Hammett et al., 2006 [11] | 06/2003 | Langson | 140 | 327 | 42.8% |
| Hammett et al., 2006 [11] | 12/2003 | Langson | 124 | 335 | 37.0% |
| Des Jarlais et al., 2007 [10] | 06/2004 | Langson | 8 | 64 | 12.5% |
| Des Jarlais et al., 2007 [10] | 12/2004 | Langson | 3 | 55 | 5.5% |
| Hammett et al., 2006 [11] | 06/2005 | Langson | 105 | 333 | 31.5% |
| Nguyen et al., 2006 [16] | 01-04/2004 | Soctrang | 80 | 341 | 23.5% |
| Nguyen et al., 2006 [16] | 01-04/2004 | Soctrang | 5 | 158 | 3.2% |
| MOH, 2006 [17] | 09-12/2004 | Angiang | 66 | 360 | 18.3% |
| MOH, 2006 [17] | 09-12/2004 | Dienbien | 183 | 359 | 51.0% |
| MOH, 2006 [17] | 09-12/2004 | Dongthap | 4 | 376 | 1.1% |
| MOH, 2006 [17] | 09-12/2004 | Kiengiang | 13 | 360 | 3.6% |
| Des Jarlais et al., 2007 [10] | 06/2005 | Langson | 91 | 337 | 27.0% |
| Nguyen et al., 2006 [16] | 03-05/2005 | Soctrang | 51 | 438 | 11.6% |
| Nguyen et al., 2006 [16] | 03-05/2005 | Soctrang | 1 | 62 | 1.6%^[[2]](#footnote-2)^ |
| Nguyen et al., 2006 [16] | 10-11/2005 | Soctrang | 23 | 250 | 9.2% |
| Quan et al., 2011 [18] | 2005-2007 | Thainguyen | 204 | 894 | 22.8% |
| Clatts et al., 2007/11* [19, 20] | 01-07/2005 | Hanoi | - | 1115 | - |
| Clatts et al., 2007 [21] | 10/2005-12/2006 | Hanoi | 22 | 179 | 12.3% |
| Clatts et al., 2007^† [22]^ | - | Hanoi | 37 | 601 | 6.2% |
| MOH, 2006 [23] | 11/2005-06/2006 | Angiang | 40 | 300 | 13.3% |
| MOH, 2006 [23] | 11/2005-06/2006 | Cantho | 110 | 299 | 36.8% |
| MOH, 2006 [23] | 11/2005-06/2006 | Danang | 5 | 260 | 1.9% |
| MOH, 2006 [23] | 11/2005-06/2006 | Haiphong | 198 | 301 | 65.8% |
| MOH, 2006 [23] | 11/2005-06/2006 | Hanoi | 71 | 296 | 24.0% |
| MOH, 2006 [23] | 11/2005-06/2006 | HCMC | 101 | 296 | 34.1% |
| MOH, 2006 [23] | 11/2005-06/2006 | Quangninh | 156 | 266 | 58.6% |
| PIHCM, 2007 [24] | 07-12/2007 | Vinhlong | 129 | 360 | 35.8% |
| PIHCM, 2009 [25] | 06-11/2008 | BRVT | 41 | 300 | 13.7% |
| PIHCM, 2009 [26] | 08-12/2008 | Soctrang | 19 | 240 | 7.9% |
| UNODC, 2009 [27] | 2009 | Dienbien | 67 | 151 | 44.4% |
| MOH, 2011 [28] | 2009 | Angiang | 47 | 300 | 15.7% |
| MOH, 2011 [28] | 2009 | Cantho | 75 | 277 | 27.1% |
| MOH, 2011 [28] | 2009 | Danang | 2 | 291 | 0.7% |
| MOH, 2011 [28] | 2009 | Dienbien | 168 | 301 | 55.8% |
| MOH, 2011 [28] | 2009 | Dongnai | 72 | 300 | 24.0% |
| MOH, 2011 [28] | 2009 | Haiphong | 144 | 300 | 48.0% |
| MOH, 2011 [28] | 2009 | Hanoi | 37 | 300 | 12.3% |
| MOH, 2011 [28] | 2009 | HCMC | 148 | 310 | 47.7% |
| MOH, 2011 [28] | 2009 | Laocai | 65 | 300 | 21.7% |
| MOH, 2011 [28] | 2009 | Nghean | 73 | 300 | 24.3% |
| MOH, 2011 [28] | 2009 | Quangninh | 167 | 300 | 55.7% |
| MOH, 2011 [28] | 2009 | Yenbai | 132 | 360 | 36.7% |
| **Street-based female sex workers** | | | | | |
| Le et al., 2000^†^ [29] | 1997 | HCMC | 5 | 172 | 2.9% |
| Nguyen et al, 2001* [6] | 06-11/2000 | Hanoi | - | 409 | - |
| Nguyen et al, 2001* [6] | 06-11/2000 | Haiphong | - | 78 | - |
| Nguyen et al, 2001* [6] | 06-11/2000 | Danang | - | 323 | - |
| Nguyen et al, 2001* [6] | 06-11/2000 | HCMC | - | 314 | - |
| Nguyen et al, 2001* [6] | 06-11/2000 | Cantho | - | 176 | - |
| Nguyen et al., 2003 [30] | 12/2000 | HCMC | 65 | 398 | 16.3% |
| Truong et al., 2004^†^ [31] | 10/12/2000 | Khanh Hoa | 0 | 132 | 0.0% |
| Nguyen et al., 2007 [8] | 04-06/2002 | Angiang | 97 | 400 | 24.3% |
| Nguyen et al., 2007 [8] | 04-06/2002 | Dienbien | 1 | 54 | 1.9% |
| Nguyen et al., 2007 [8] | 04-06/2002 | Dongthap | 3 | 201 | 1.5% |
| Nguyen et al., 2007 [8] | 04-06/2002 | Kiengiang | 0 | 216 | 0.0% |
| MOH, 2002 [32] | 04-06/2002 | Quangtri | 1 | 13 | 7.7% |
| MOH, 2006 [17] | 09-12/2004 | Angiang | 38 | 325 | 11.7% |
| MOH, 2006 [17] | 09-12/2004 | Dienbien | 1 | 26 | 3.8% |
| MOH, 2006 [17] | 09-12/2004 | Dongthap | 1 | 181 | 0.6% |
| MOH, 2006 [17] | 09-12/2004 | Kiengiang | 8 | 330 | 2.4% |
| MOH, 2006 [17] | 09-12/2004 | Quangtri | 1 | 17 | 5.9% |
| MOH, 2006 [23] | 11/2005-06/2006 | AnGiang | 12 | 238 | 5.0% |
| MOH, 2006 [23] | 11/2005-06/2006 | Cantho | 47 | 162 | 29.0% |
| MOH, 2006 [23] | 11/2005-06/2006 | Danang | 1 | 175 | 0.6% |
| MOH, 2006 [23] | 11/2005-06/2006 | Haiphong | 20 | 279 | 7.2% |
| MOH, 2006 [23] | 11/2005-06/2006 | Hanoi | 62 | 275 | 22.5% |
| MOH, 2006 [23] | 11/2005-06/2006 | HCMC | 33 | 298 | 11.1% |
| MOH, 2006 [23] | 11/2005-06/2006 | Quangninh | 20 | 161 | 12.4% |
| MOH, 2011 [28] | 2009 | AnGiang | 23 | 300 | 7.7% |
| MOH, 2011 [28] | 2009 | Cantho | 27 | 138 | 19.6% |
| MOH, 2011 [28] | 2009 | Danang | 1 | 300 | 0.3% |
| MOH, 2011 [28] | 2009 | Dongnai | 14 | 300 | 4.7% |
| MOH, 2011 [28] | 2009 | Haiphong | 69 | 300 | 23.0% |
| MOH, 2011 [28] | 2009 | Hanoi | 59 | 300 | 19.7% |
| MOH, 2011 [28] | 2009 | HCMC | 49 | 299 | 16.4% |
| MOH, 2011 [28] | 2009 | Nghean | 9 | 282 | 3.2% |
| MOH, 2011 [28] | 2009 | Quangninh | 2 | 159 | 1.3% |
| MOH, 2011 [28] | 2009 | Yenbai | 16 | 151 | 10.6% |
| **Entertainment-based female sex workers** | | | | | |
| Truong et al., 2004^†^ [31] | 10/12/2000 | Khanh Hoa | 0 | 52 | 0.0% |
| Nguyen et al, 2001* [6] | 06-11/2000 | Hanoi | - | 480 | - |
| Nguyen et al, 2001* [6] | 06-11/2000 | Haiphong | - | 504 | - |
| Nguyen et al, 2001* [6] | 06-11/2000 | Danang | - | 449 | - |
| Nguyen et al, 2001* [6] | 06-11/2000 | HCMC | - | 463 | - |
| Nguyen et al, 2001* [6] | 06-11/2000 | Cantho | - | 406 | - |
| Nguyen et al., 2007 [8] | 04-06/2002 | AnGiang | 60 | 370 | 16.2% |
| Nguyen et al., 2007 [8] | 04-06/2002 | Dienbien | 0 | 49 | 0.0% |
| Nguyen et al., 2007 [8] | 04-06/2002 | Dongthap | 4 | 291 | 1.4% |
| Nguyen et al., 2007 [8] | 04-06/2002 | Kiengiang | 6 | 449 | 1.3% |
| MOH, 2002 [32] | 04-06/2002 | Quangtri | 1 | 152 | 0.7% |
| MOH, 2006 [17] | 09-12/2004 | AnGiang | 29 | 488 | 5.9% |
| MOH, 2006 [17] | 09-12/2004 | Dongthap | 7 | 365 | 1.9% |
| MOH, 2006 [17] | 09-12/2004 | Kiengiang | 5 | 432 | 1.2% |
| MOH, 2006 [17] | 09-12/2004 | Quangtri | 0 | 254 | 0.0% |
| MOH, 2006 [23] | 11/2005-06/2006 | AnGiang | 39 | 361 | 10.8% |
| MOH, 2006 [23] | 11/2005-06/2006 | Cantho | 7 | 300 | 2.3% |
| MOH, 2006 [23] | 11/2005-06/2006 | Danang | 3 | 313 | 1.0% |
| MOH, 2006 [23] | 11/2005-06/2006 | Haiphong | 14 | 274 | 5.1% |
| MOH, 2006 [23] | 11/2005-06/2006 | Hanoi | 21 | 224 | 9.4% |
| MOH, 2006 [23] | 11/2005-06/2006 | HCMC | 18 | 302 | 6.0% |
| MOH, 2006 [23] | 11/2005-06/2006 | Quangninh | 8 | 185 | 4.3% |
| MOH, 2011 [28] | 2009 | AnGiang | 8 | 263 | 3.0% |
| MOH, 2011 [28] | 2009 | Cantho | 12 | 354 | 3.4% |
| MOH, 2011 [28] | 2009 | Danang | 1 | 251 | 0.4% |
| MOH, 2011 [28] | 2009 | Dongnai | 7 | 300 | 2.3% |
| MOH, 2011 [28] | 2009 | Haiphong | 35 | 300 | 11.7% |
| MOH, 2011 [28] | 2009 | Hanoi | 53 | 300 | 17.7% |
| MOH, 2011 [28] | 2009 | HCMC | 49 | 304 | 16.1% |
| MOH, 2011 [28] | 2009 | Nghean | 3 | 274 | 1.1% |
| MOH, 2011 [28] | 2009 | Quangninh | 8 | 298 | 2.7% |
| MOH, 2011 [28] | 2009 | Yenbai | 6 | 123 | 4.9% |
| **Unspecified-based female sex workers** | | | | | |
| Le et al., 2010* [33] | 12/1996-06/1997 | HCMC | - | 310 | - |
| Grayman et al., 2005* [34] | 10/12/2000 | Khanh Hoa | - | 610 | - |
| Tran et al., 2007 [35] | 06-09/2002 | Hanoi | 47 | 400 | 11.8% |
| Tran et al., 2007 [36] | 06-09/2002 | Hanoi | - | 400 | 11.8% |
| Tran et al., 2007 [37] | 06-09/2002 | Hanoi | - | 400 | 11.8% |
| Nguyen et al., 2009 [38] | 05-08/2003 | Soctrang | 13 | 395 | 3.3% |
| Nguyen et al., 2008 [39] | 05-08/2003 | Soctrang | - | 395 | - |
| Thuong et al., 2005 [40] | 12/2002-02/2003 | Angiang | 21 | 300 | 7.0% |
| Thuong et al., 2005 [40] | 12/2002-02/2003 | Dongthap | 7 | 149 | 4.7% |
| Thuong et al., 2005 [40] | 12/2002-02/2003 | Kiengiang | 10 | 253 | 4.0% |
| Thuong et al., 2005 [40] | 12/2002-02/2003 | LaiChau | 2 | 100 | 2.0% |
| Thuong et al., 2005 [40] | 12/2002-02/2003 | Quangtri | 1 | 101 | 1.0% |
| Nguyen et al., 2005* [41] | 12/2002-02/2003 | 5 provinces | - | 904 | - |
| O’Farrell et al., 2006* [42] | 12/2002-02/2003 | 5 provinces | - | 904 | - |
| Vu Thuong et al., 2007 [43] | 11-12/2004 | Angiang | 15 | 284 | 5.3% |
| Vu Thuong et al., 2007 [43] | 09-12/2004 | Dienbien | 4 | 112 | 3.6% |
| Vu Thuong et al., 2007 [43] | 11-12/2004 | Dongthap | 5 | 196 | 2.6% |
| Vu Thuong et al., 2007 [43] | 11-12/2004 | Kiengiang | 12 | 293 | 4.1% |
| Vu Thuong et al., 2007 [43] | 11-12/2004 | LaiChau | 2 | 99 | 2.0% |
| Vu Thuong et al., 2007 [43] | 11-12/2004 | Quangtri | 1 | 100 | 1.0% |
| Johnston et al., 2008 [44] | 04-06/2004 | Haiphong | 73 | 215 | 34.0% |
| Johnston et al., 2008 [44] | 05-07/2004 | HCMC | 59 | 413 | 14.3% |
| Nemoto et al., 2008 [45] | 11/2004-1/2005 | HCMC | 11 | 159 | 6.9% |
| **Men who have sex with men** | | | | | |
| Colby et al., 2003^†^ [46] | 04-05/2001 | HCMC | 3 | 66 | 4.5% |
| Clatts et al., 2007 [19] | 2002 | Hanoi | 23 | 79 | 29.1% |
| Nguyen et al., 2008 [47] | 04-05/2004 | HCMC | 47 | 599 | 7.8% |
| Colby et al., 2008 [48] | 07-12/2005 | Khanhhoa | 0 | 295 | 0.0% |
| MOH, 2006 [17] | 12/2005-6/2006 | Hanoi | 37 | 397 | 9.3% |
| MOH, 2006 [17] | 12/2005-6/2006 | HCMC | 21 | 393 | 5.3% |
| MOH, 2011 [28] | 2009 | Hanoi | 69 | 399 | 17.3% |
| MOH, 2011 [28] | 2009 | Haiphong | 66 | 400 | 16.5% |
| MOH, 2011 [28] | 2009 | HCMC | 59 | 398 | 14.8% |
| MOH, 2011 [28] | 2009 | Cantho | 24 | 398 | 6.0% |
| Pham et al., 2012 [49] | 08-12/2009 | Angiang | 24 | 381 | 6.3% |
| **Other groups** | | | | | |
| Nguyen et al., 2009 [2] | 04/2007 (clients of FSWs) | Haiphong | 13 | 292 | 4.5% |
| Hammett et al., 2010 [50] | 04-05/2008 (partner of PWID) | Hanoi | 32 | 232 | 13.8% |
| Hammett et al., 2011 [51] | 06/2009 ( partner of PWID) | Hanoi | 27 | 291 | 9.3% |
| Hammett et al., 2011 [51] | 08/2010 (partner of PWID) | Hanoi | 18 | 278 | 6.5% |

FSWs, female sex workers,; PWID, people who inject drugs; HCMC, Ho Chi Minh City; MOH, Ministry of Health; n, frequency; N, total participants.

*Articles only reported levels of risk-taking behaviours, access to HIV testing services, and/or sexually transmitted infections. Required data may be reported in different articles that used the same dataset of a study.

† This is the high prevalence of self-reported HIV status.

**Fig B. HIV prevalence among various populations in Vietnam, 2000-2011.** Data source for people who inject drugs (PWID) [4, 5, 7-13, 16-18, 21-26, 28, 52], men who have sex with men (MSM) [19, 23, 28, 46-49, 52], street-based female sex workers (FSWs) [8, 17, 23, 28, 29, 31, 32, 52], entertainment-based FSWs [8, 17, 23, 28, 31, 32, 52], clients [2, 52], and low-risk populations [52].

**HIV prevalence (%)**

**HIV prevalence (%)**

**Fig C. Number of reported HIV diagnoses in Vietnam, 2000-2011.**

**Fig D. Number of PWID registered methadone maintenance therapy.**

**Year**

**Fig E. Number of people receiving ART in Vietnam, 2006-2010.**

**Number of people**

**Year**

***HIV programme spending in Vietnam during 2006-2010***

Available data on HIV spending in Vietnam in 2006-2010 were used. Extracting relevant data from the latest report of the National AIDS Spending Assessment (NASA) was applied for the period 2008-2010 [53]. Through a local data team and comprehensive online searches, we obtained data on total HIV spending of the Government of Vietnam, and five key donors including the United States President's Emergency Plan for AIDS Relief, World Bank, United Kingdom Aid from the Department for International Development, Global Fund to fight AIDS, Tuberculosis and Malaria, and Asian Development Bank in 2006-2007. According to the NASA report [53], HIV spending in 2008-2010 was contributed by the Vietnamese government (14.3%), the private sector (15.1%) and the international sources (70.6%). Among international sources, 88.9% of the total spending was contributed by the five aforementioned major donors. Thereafter, we used these percentages to estimate the investment from small international donors and the private sector for the period 2006-2007. Our spending estimates were US$117,489,961 during 2006-2007, highly comparable to official government’s figures (US$116,280,815 over 2006-2007 [53, 54]). The spending breakdown of total budgets to programme areas and supporting costs was done by using proportional allocations available in the period 2008-2010, as shown in Table B.

Table B. Estimated HIV spending in Vietnam, 2006-2010.

| **Funding allocations** | **Cost (US$)** | | | | |
| --- | --- | --- | --- | --- | --- |
|  | **2006** | **2007** | **2008** | **2009** | **2010** |
| **Total** | 48,011,375 | 69,478,586 | 96,208,777 | 127,374,483 | 139,253,245 |
| **Donor** |  |  |  |  |  |
| PEPFAR | 18,937,600 | 24,006,400 | 38,894,158 | 63,926,353 | 69,340,357 |
| DFID/WB | 10,509,160 | 11,089,142 | 10,018,210 | 10,700,301 | 9,383,343 |
| Asian Development Bank | 870,000 | 7,730,000 | 6,251,409 | 6,320,161 | 6,152,088 |
| Global Fund | 1,817,577 | 2,266,386 | 2,871,788 | 5,829,561 | 6,650,517 |
| Government | 5,947,233 | 10,176,357 | 13,459,880 | 17,176,061 | 21,431,087 |
| Private sector | 6,298,625 | 9,114,913 | 16,014,322 | 16,036,519 | 15,600,379 |
| Others | 3,631,180 | 5,095,388 | 8,699,010 | 7,385,527 | 10,695,474 |
| **Programmatic spending** |  |  |  |  |  |
| Direct costs for prevention | 15,571,232 | 22,533,559 | 31,913,529 | 40,811,053 | 44,951,932 |
| Direct costs for care and treatment | 13,207,797 | 19,113,368 | 24,274,597 | 33,378,767 | 42,161,961 |
| Indirect costs | 19,232,346 | 27,831,659 | 40,020,651 | 53,184,663 | 52,139,352 |
| **HIV prevention costs** |  |  |  |  |  |
| Mass information, education and communication | 2,683,490 | 3,883,352 | 6,861,283 | 7,423,982 | 5,994,680 |
| NSPs for PWID | 2,477,814 | 3,585,713 | 4,624,843 | 5,655,741 | 7,591,711 |
| MMT for PWID |  |  | 77,538 | 118,828 | 656,930 |
| Programmes for FSWs/clients | 1,276,363 | 1,847,060 | 3,021,373 | 3,917,357 | 2,707,129 |
| Programmes for MSM | 383,729 | 555,305 | 835,613 | 1,007,369 | 1,056,975 |
| VCT programmes | 1,120,728 | 1,621,837 | 1,308,824 | 2,483,879 | 4,676,980 |
| STI programme (including microbicides) | 174,501 | 252,525 | 253,540 | 287,499 | 777,716 |
| PMTCT | 933,685 | 1,351,162 | 1,673,181 | 2,402,482 | 2,980,480 |
| Blood safety | 1,509,227 | 2,184,043 | 4,316,317 | 3,965,649 | 3,123,716 |
| Prevention for PLHIV | 224,864 | 325,407 | 234,779 | 548,978 | 915,607 |
| Prevention for youth/people in work force/vulnerable and accessible populations/ healthcare workers | 1,410,596 | 2,041,313 | 3,467,647 | 2,714,047 | 4,166,602 |
| Programmes for unspecified groups | 80,330 | 116,247 | 49,126 | 204,681 | 665,276 |
| Unclassified/not disaggregated | 3,295,905 | 4,769,596 | 5,189,465 | 10,080,561 | 9,638,130 |
| **HIV care and treatment costs** |  |  |  |  |  |
| Antiretroviral therapy | 3,603,531 | 5,214,769 | 6,172,042 | 8,118,648 | 12,942,285 |
| OI prophylaxis and treatment | 2,178,302 | 3,152,280 | 4,617,644 | 5,061,769 | 6,782,672 |
| Home-based HIV care | 2,597,979 | 3,759,607 | 6,013,968 | 7,033,055 | 6,586,691 |
| Other im-/out-patient HIV care | 917,841 | 1,328,233 | 1,243,154 | 1,706,885 | 3,986,365 |
| Unclassified/not disaggregated | 3,910,144 | 5,658,478 | 6,227,789 | 11,458,410 | 11,863,948 |
| **Indirect costs** |  |  |  |  |  |
| Program management and administration strengthening | 13,827,517 | 20,010,181 | 28,980,635 | 38,745,169 | 36,772,929 |
| Human resources | 3,214,820 | 4,652,255 | 6,600,136 | 7,999,570 | 9,695,665 |
| Enabling environment | 1,129,960 | 1,635,197 | 2,574,831 | 3,444,920 | 2,519,703 |
| Social protection and services (including services for OVC) | 655,786 | 949,006 | 1,158,137 | 1,786,977 | 2,010,858 |
| Research | 404,263 | 585,020 | 706,912 | 1,208,027 | 1,140,197 |

DFID, United Kingdom Department for International Development FSWs, female sex workers; MMT, methadone maintenance therapy; MSM, men who have sex with men; NSP, needle syringe programme; OI, opportunistic infection; OVC, orphan vulnerable children; PEPFAR, United States President's Emergency Plan for AIDS Relief; PLHIV, people living. with HIV; PMTCT; prevention mother-to-child transmission of HIV; STI, sexually transmitted infection; PWID, people who inject drugs; VCT, voluntary HIV counselling and testing; WB, World Bank.

1. Description of Mathematical Model

We investigated the cost effectiveness of HIV prevention programmes in Vietnam and the impact of changes in HIV funding using a detailed mathematical model of HIV transmission. Relating the changes in funding to the appropriate transmission parameters in the model, we calculated the change in HIV incidence, the number of HIV/AIDS deaths and cost-effectiveness of HIV prevention programmes. To do this we used the Optima, which was developed from the HIV in Indonesia Model (HIM). Previously, HIM was used to investigate the impact of HIV prevention programmes in eight regions of Indonesia. A detailed description of Optima is provided as follows [55].

Informed by available HIV surveillance data the model divides the 15-49 year-old population in Vietnam into seven distinct population groups as follows: low-risk males (LRM), low-risk females (LRF), direct and indirect female sex workers (DFSWs and IFSWs), clients of sex workers (CSWs), MSM, and (PWID). The model scheme is summarised in Fig. F.

Optima was specifically calibrated for Vietnam using best-practice HIV epidemic modelling techniques incorporating realistic biological transmission processes, detailed infection progression, and sexual mixing patterns and drug injection behaviours. Through a set of ordinary differential equations, the model tracks HIV transmission and the number of HIV positive people and their rate of disease progression via CD4 T-cell count. The *Optima* also records deaths due to HIV/AIDS or other causes. The model distinguishes people who are undiagnosed, diagnosed, and on effective ART as shown in Fig. F. HIV transmission within the population occurs through the interaction between different population groups (Table C). HIV infections occur through regular, casual, or commercial sexual partnerships or through sharing of injecting equipment.

**Table C. Population interactions.**

| **Population** | **LRM** | **LRF** | **DFSWs** | **IFSWs** | **CSWs** | **MSM** | **PWID** |
| --- | --- | --- | --- | --- | --- | --- | --- |
| **LRM** |  | RC | RC | RC |  |  |  |
| **LRF** |  |  |  |  |  |  |  |
| **DFSWs** |  |  |  |  |  |  |  |
| **IFSWs** |  |  |  |  |  |  |  |
| **CSWs** |  | RC | RC$ | RC$ |  |  |  |
| **MSM** |  | R | R | R |  | C$ |  |
| **PWID** |  | RC | RC$ | RC$ |  |  | I |

Notes: R, regular sex; C, casual sex; $, commercial sex; I, injecting. Rows show insertive acts and columns show receptive acts

Sexual transmission depends on the prevalence of HIV, the number of casual and regular homosexual and heterosexual partnerships per person, the frequency of sexual acts within a partnership, condom usage, male circumcision, and the infection stage of HIV-positive partners. For PWID, intravenous transmission is dependent on number of injecting partners, frequency of injecting, frequency of sharing equipment, cleaning of syringes, and the efficacy of cleaning. These factors are incorporated into risk-equations within the model to determine the annual per-capita risk of a susceptible person becoming infected with HIV. Optima describes the impact of HIV prevention programmes indirectly through their influence on behavioural, clinical, and injecting parameters. MMT is explicitly incorporated into the model.

Model input parameters were informed by all available behavioural data regarding sexual or injecting risk activities, biological data on disease progression and heterogeneous transmission rates, and clinical data (such as rates of HIV testing and antiretroviral coverage). Any data available from 2000-2011 were used as inputs; where data were not available, assumptions were made based on consultations with Vietnamese stakeholders.

To calibrate Optima to the HIV epidemic, all parameters were first assigned a best estimate with uncertainty bounds. The model was then calibrated using adaptive stochastic linear gradient-descent optimization to identify parameter values that yielded epidemic projections that matched available population-level epidemiological data from 2000-2011 including HIV prevalence in each population group, the number of diagnoses, and the number of people on ART.

The model uses a coupled system of ordinary differential equations to track the movement of people between health states. The overall population is partitioned in two ways: by group and by health state. Individuals are assigned to a given population based on their dominant risk; however, to capture important cross-modal types of transmission (e.g., FSW becoming infected via injecting drug use), relevant behavioural parameters can be set to small but nonzero values (e.g., male PWID occasionally engage in commercial sex; CSW occasionally inject drugs).

The rate at which uninfected individuals in each population group become infected is determined by the force-of-infection for that population. This depends on the number of risk events an individual is exposed to in a given period of time and the infection probability of each event. Sexual transmission risk depends on the number of people in each HIV-infected stage (that is, the prevalence of infection in the population of partners), the average number of casual, regular, and commercial homosexual and heterosexual partnerships per person, the average frequency of sexual acts per partnership, the proportion of these acts in which condoms are used, the efficacy of condoms, the extent of male circumcision, and the prevalence levels of ulcerative sexually transmitted infections (which increase transmission probability) and HIV. The stage of infection (chronic, AIDS-related illness/late stage, or on treatment) for the HIV-positive partner in a serodiscordant couple also influences transmission risk due to different levels of infectiousness in each infection stage. Intravenous transmission risk depends on the number of injecting partners per person per year, frequency of injecting per year, frequency of sharing injecting equipment and percentage of shared syringes that are cleaned before re-use and the efficacy of cleaning.

Mathematically, the force-of-infection is given by:

$$\lambda=1-\left( 1-\beta\right)^{n}$$

where $\lambda$ is the force-of-infection, $\beta$ is the transmission probability of each event, and *n* is the effective number of at-risk events (thus *n* gives the average number interaction events with infected people where HIV transmission may occur). The value of the transmission probability $\beta$ is inversely related to CD4 count, differs for different modes of transmission (intravenous drug injection, heterosexual intercourse, and homosexual intercourse), and may be modified by behavioural interventions (for example, condom use or circumcision). The number of events *n* not only incorporates the total number of events, but also other factors that moderate the possibility that these events are capable of transmitting infection, such as condom use or circumcision. There is one force-of-infection term for each type of interaction (for example, casual sexual relationships between low-risk males and indirect female sex workers), and the force-of-infection for a given population will be the sum of overall interaction types.

In addition to the force-of-infection rate, in which individuals move from uninfected to infected states, there are seven other means by which individuals may move between health states. First, individuals may die, either due to the background death rate (which affects all populations equally), due to injecting behaviour, or due to HIV/AIDS (which depends on CD4 count). Second, in the absence of intervention, individuals progress from higher to lower CD4 counts. Third, individuals can move from undiagnosed to diagnosed states based on their HIV testing rate, which is a function of CD4 count (for example, people with AIDS symptoms have a higher testing rate) and population type (for example, PWID usually get tested more frequently than low-risk males). Fourth, diagnosed individuals may move onto treatment, at a rate which is dependent on CD4 count. Fifth, individuals may move from treatment to treatment failure, and sixth, from treatment failure onto second-line treatment. Finally, while on successful first- or second-line treatment, individuals may progress from lower to higher CD4 count.

In total, the model can accommodate up to 294 compartments (14 populations each with 21 health states), and the change in the number of people in each compartment is determined by the sum over the relevant rates described above multiplied by the compartments on which they act. For example, the number of individuals in the compartment corresponding to undiagnosed FSWs with a CD4 count between 200 and 350 cells/µL changes according to the following equation:

$$\frac{dU_{FSW200-350}}{dt}={U_{FSW}}_{350-500}\tau_{350-500}-{U_{FSW}}_{200-350}\left( \mu_{200-350}+\tau_{200-350}+{\eta_{FSW}}_{350-500} \right)$$

where ${U_{FSW}}_{350-500}$ is the current population size of people with undiagnosed HIV and with a CD4 count between 350 and 500 cells/μL, ${U_{FSW}}_{200-350}$ is the population size of the compartment with lower CD4 count (200-350 cells/μL), $\tau$ is the disease progression rate for the given CD4 count, $\mu$ is the death rate, and $\eta$ is the HIV testing rate. (Note: this example does not consider movement between populations, such as FSWs returning to the low-risk female population and vice versa). Each compartment (Fig. F, boxes) corresponds to a single differential equation in the model, and each rate (Fig. F, arrows) corresponds to a single term in that equation.

**Fig F. Model schematic for HIV infection progression.**


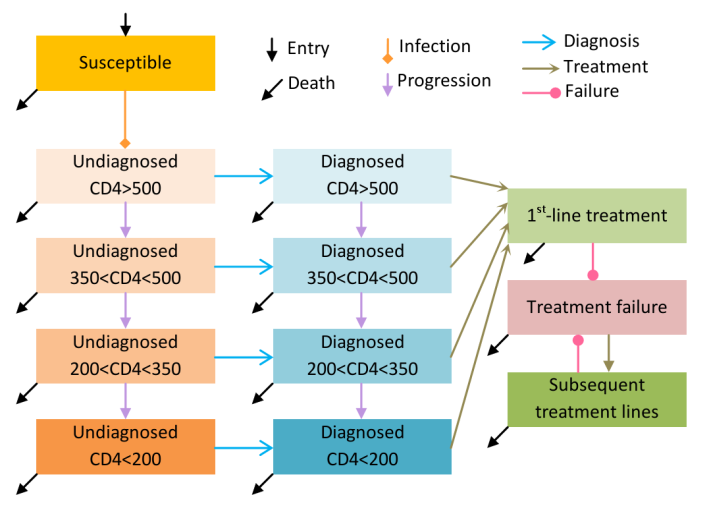


1. Model Parameters

Table D represents model inputs at national level. Main data sources include:

1. 2000 HIV/AIDS Behavioural Surveillance Survey (BSS 2000).
2. 2002 and 2004 Baseline and Endpoint Survey Report of ADB Project.
3. 2005-2006 HIV/STI Integrated Biological and Behavioural Surveillance (IBBS 2006).
4. 2009 HIV/STI Integrated Biological and Behavioural Surveillance (IBBS 2009)
5. Estimated size of at-risk population by the Vietnam Authority of HIV/AIDS Control.
6. General Statistics Office of Vietnam.

**Table D. Mathematical modelling inputs.** In this table, behavioural estimates from cross-sectional surveys among a given population in part of the country are assumed to be representative that for the entire population in the country and are comparable over time. As average data, specifically quantitative variables (e.g., number of injection or sexual acts) were extracted from reports without information on variables’ actual distributions, they are assumed to be normally distributed and thus the mean (with range) is used as model inputs for continuous variables**.**

| **Parameters** | **2000** | **2001** | **2002** | **2003** | **2004** | **2005** | **2006** | **2007** | **2008** | **2009** | **2010** | **Ref** |
| --- | --- | --- | --- | --- | --- | --- | --- | --- | --- | --- | --- | --- |
| **Low risk males (excluding men who do not inject drugs, clients of female sex workers, and men who have sex with men)** | | | | | | | | | | | |  |
| Estimated population size (in thousand [min-max] |  |  |  |  |  |  |  |  |  | 28,803  (27,759–29,782 |  | [56, 57] |
| Average number of regular sexual acts per year |  |  |  |  |  | 50  (38–63) |  |  |  |  |  | [58] |
| Average number of casual sexual acts per year |  |  |  |  |  | 1  (1–1) |  |  |  |  |  | [58] |
| Condom use percentage for last sex act with regular partner |  |  |  |  |  | 19.2%  (17.1–21.5%) |  |  | 22.6%  (20.9–24.2%) |  |  | [59, 60] |
| Condom use percentage for last sex act with casual partner |  |  |  |  |  | 75.5%  (62.5–79.1%) |  |  |  |  |  | [59, 60] |
| Circumcision probability | 5.0%  (2.0–6.0%) |  |  |  |  |  |  |  |  |  |  | (Experienced estimate) |
| HIV testing rate per year |  |  |  |  |  | 2.6%  (2.2–3.0%) |  |  |  |  |  | [58] |
| **Male clients of female sex workers** | | | | | | | | | | | |  |
| Estimated population size (in thousand [min-max] |  |  |  |  |  |  |  |  |  | 2,372  (1,582–3,163) |  | [57] |
| Average number of regular sexual acts per year |  |  |  |  |  |  |  | 98  (73–122) |  |  |  | [2] |
| Average number of casual sexual acts per year |  |  |  |  |  |  |  | 34  (26–43) |  |  |  | [2] |
| Condom use percentage for last sex act with regular partner |  |  |  |  |  |  |  | 29.4%  (22.9–36.9%) |  |  |  | [2] |
| Condom use percentage for last sex act with casual partner |  |  |  |  |  |  |  | 94.5%  (91.3–96.6%) |  |  |  | [2] |
| Circumcision probability | 5.0%  (2.0–6.0%) |  |  |  |  |  |  |  |  |  |  | (Experienced estimate) |
| Average number of injections per year |  |  |  |  |  |  |  | 28  (21–35) |  |  |  | [2] |
| HIV testing rate per year |  |  |  |  |  |  |  | 18.5%  (14.5–23.3%) |  |  |  | [2] |
| Prevalence of syphilis |  |  |  |  |  |  |  | 1.37%  (0.53–3.46%) |  |  |  | [2] |
| **People who inject drugs** |  |  |  |  |  |  |  |  |  |  |  |  |
| Estimated population size (in thousand [min-max] |  |  |  |  |  |  |  |  |  | 217  (98–335) |  | [57] |
| Average number of regular sexual acts per year | 28  (21–35) |  | 35  (26–44) |  | 27  (20–35) |  | 48  (36–60) |  |  | 60  (45–75) |  | [17, 23, 28, 32, 61] |
| Average number of casual sexual acts per year | 2  (2–3) |  | 2  (2–3) |  | 2  (2–3) |  | 3  (2–4) |  |  | 2  (2–3) |  | [17, 23, 28, 32, 61] |
| Average number of commercial sexual acts per year | 4  (3–5) |  | 9  (7–11) |  | 6  (5–8) |  | 5  (4–6) |  |  | 5  (4–6) |  | [17, 23, 28, 32, 61] |
| Condom use percentage for last sex act with regular partner | 32.3%  (28.5–36.1%) |  | 28.7%  (26.2–31.4%) |  | 44.0%  (40.4–47.7%) |  | 35.5%  (32.5–38.5%) |  |  | 50.7%  (48.5–52.9%) |  | [17, 23, 28, 32, 61] |
| Condom use percentage for last sex act with casual partner | 50.4%  (42.5–58.3%) |  | 46.8%  (42.8–50.5%) |  | 62.2%  (58.6–65.8%) |  | 45.6%  (39.8–51.3%) |  |  | 57.6%  (52.1–62.1%) |  | [17, 23, 28, 32, 61] |
| Condom use percentage for last sex act with commercial partner | 74.7%  (69.1–80.1%) |  | 62.9%  (60.0–65.9%) |  | 86.6%  (84.1–89.2%) |  | 66.6%  (62.7–70.5%) |  |  | 78.7%  (76.0–81.5%) |  | [17, 23, 28, 32, 61] |
| Circumcision probability | 10.0%  (8.0-12.0%) |  |  |  |  |  |  |  |  |  |  | [62] |
| Average number of injections per year | 562  (511–851) |  | 514  (385–642) |  | 217  (162–642) |  | 684  (513–855) |  |  | 637  (478–796) |  | [17, 23, 28, 32, 61] |
| Percentage of shared injections |  |  | 22.8%  (21.3–24.3%) |  | 16.1%  (14.1–18.1%) |  | 17.8%  (16.1–19.4%) |  |  | 12.5%  (11.4–13.6%) |  | [17, 23, 28, 32] |
| Percentage of reused syringes that are cleaned |  |  |  |  | 4.8%  (2.1–10.7%) | 11.9%  (7.7–18.1%) |  |  |  |  |  | [16] |
| HIV testing rate per year |  |  |  |  |  |  | 8.9%  (7.6–10.1%) |  |  | 20.3%  (19.0–21.6%) |  | [23, 28] |
| Prevalence of syphilis |  |  |  |  |  |  | 1.8%  (1.3–2.5%) |  |  | 0.8%  (0.6–1.2%) |  | [23, 28] |
| **Men who have sex with men** |  |  |  |  |  |  |  |  |  |  |  |  |
| Estimated population size (in thousand [min-max] |  |  |  |  |  |  |  |  |  | 385  (177–393) |  | [57] |
| Average number of sexual acts with non-commercial female partners per year |  |  |  |  |  |  | 25  (19–31) |  |  | 29  (22–36) |  | [23, 28] |
| Average number of sexual acts with non-commercial male partners per year |  |  |  |  |  |  | 52  (39–65) |  |  | 47  (35–59) |  | [23, 28] |
| Average number of sexual acts with commercial male partners per year |  |  |  |  |  |  | 29  (22–36) |  |  | 45  (34–56) |  | [23, 28] |
| Condom use percentage for last sex act with non-commercial female partner |  |  |  |  |  |  | 43.7%  (37.3–50.1%) |  |  | 43.0%  (39.1–46.8%) |  | [23, 28] |
| Condom use percentage for last sex act with non-commercial male partner |  |  |  |  | 35.7%  (29.4-42.0%) |  | 63%  (58.5–67.5%) |  |  | 56.1%  (53.0–59.3%) |  | [23, 28, 47] |
| Condom use percentage for last sex act with commercial male partner |  |  |  |  | 50.0% (35.2-64.8%) |  | 57.5%  (48.4–66.6%) |  |  | 58.9%  (52.8–65.0%) |  | [23, 28] |
| Circumcision probability | 10.0%  (8.0-12.0%) |  |  |  |  |  |  |  |  |  |  | [62] |
| Average number of injections per year |  |  |  |  |  |  | 23  (22–24) |  |  | 20  (19–21) |  | [23, 28] |
| Percentage of shared injections |  |  |  |  |  |  | 29.1%  (17.1–41.1%) |  |  | 23.3%  (15.8–30.7%) |  | [23, 28] |
| HIV testing rate per year |  |  |  |  |  |  | 5.0%  (3.4–6.5%) |  |  | 21.9%  (20.1–23.8%) |  | [23, 28] |
| Prevalence of sexually transmitted infections |  |  |  |  |  |  | 22.9%  (20.0–25.8%) |  |  | 13.6%  (12.1–15.1%) |  | [23, 28] |
| **Low risk females (excluding female sex workers)** | | | | | | | | | | | |  |
| Estimated population size (in thousand [min-max] |  |  |  |  |  |  |  |  |  | 32,960  (32,918–33,002) |  | [56, 57] |
| Average number of regular sexual acts per year |  |  |  |  |  | 52  (36–65) |  |  |  |  |  | [60] |
| Average number of casual sexual acts per year |  |  |  |  |  | 0  (n/a) |  |  |  |  |  | [60] |
| Condom use percentage for last regular sex act |  |  |  |  |  | 14.5%  (12.9–16.3%) |  |  |  |  |  | [60] |
| Condom use percentage for last casual sex act |  |  |  |  |  | 7.1%  (0.8–22.7%) |  |  |  |  |  | [60] |
| HIV testing rate per year |  |  |  |  |  | 2.1%  (1.8–2.5%) |  |  |  |  |  | [60] |
| **Street-based female sex worker*** |  |  |  |  |  |  |  |  |  |  |  |  |
| Estimated population size (in thousand [min-max] |  |  |  |  |  |  |  |  |  | 29  (13–45) |  | [57] |
| Average number of non-commercial sexual acts per year |  |  |  |  |  |  | 36  (29–43) |  |  | 29  (22–35) |  | [23, 28] |
| Average number of commercial sexual acts per year | 658  (558–728) |  | 664  (580–748) |  | 424  (308–540) |  | 655  (558–745) |  |  | 834  (753–916) |  | [17, 23, 28, 32, 61] |
| Condom use percentage for last sex act with non-commercial partner | 31.6%  (23.7–39.5%) |  | 34.2%  (31.7–36.8%) |  | 60.5%  (56.4–64.6%) |  | 41.3%  (37.7–44.8%) |  |  | 40.4%  (37.4–53.7%) |  | [17, 23, 28, 32, 61] |
| Condom use percentage for last sex act with commercial partner | 88.5%  (86.9–90.1%) |  | 81.4%  (79.7–83.1%) |  | 90.3%  (87.9–92.7%) |  | 92.1%  (90.6–93.6%) |  |  | 87.5%  (86.1–89.0%) |  | [17, 23, 28, 32, 61] |
| Average number of injections per year |  |  |  |  |  |  | 49  (37–61) |  |  | 57  (43–71) |  | [23, 28] |
| Percentage of shared injections |  |  |  |  |  |  | 25.6%  (18.1–33.3%) |  |  | 13.6%  (8.7–18.5%) |  | [23, 28] |
| HIV testing rate per year |  |  |  |  |  |  | 7.4%  (6.1–8.7%) |  |  | 26.5%  (24.8–28.2%) |  | [23, 28] |
| Prevalence of sexually transmitted infections |  |  | 22.8%  (18.6–26.9%) |  | 17.8%  (14.3–21.4%) |  | 7.1%  (5.9–8.4%) |  |  | 3.4%  (2.7–4.1%) |  | [23, 28, 43] |
| **Entertainment-based female sex worker*** |  |  |  |  |  |  |  |  |  |  |  |  |
| Estimated population size (in thousand [min-max] |  |  |  |  |  |  |  |  |  | 36  (16–56) |  | [57] |
| Average number of non-commercial sexual acts per year |  |  |  |  |  |  | 36  (30–42) |  |  | 34  (28–40) |  | [23, 28] |
| Average number of commercial sexual acts per year | 377  (325–431) |  | 327  (238–417) |  | 365  (286–440) |  | 591  (521–661) |  |  | 730  (661–801) |  | [17, 23, 28, 32, 61] |
| Condom use percentage for last sex act with non-commercial partner | 48.6%  (36.5–60.8%) |  | 45.3%  (42.3–48.2%) |  | 60.1%  (57.2–62.9%) |  | 37.8%  (34.8–62.9%) |  |  | 40.1%  (37.5–42.7%) |  | [17, 23, 28, 32, 61] |
| Condom use percentage for last sex act with commercial partner | 90.3%  (89.1–91.5%) |  | 69.6%  (66.9–72.3%) |  | 85.6%  (83.6–87.7%) |  | 93.4%  (92.2–94.6%) |  |  | 88.0%  (86.7–89.3%) |  | [17, 23, 28, 32, 61] |
| Average number of injections per year |  |  |  |  |  |  | 19  (14–24) |  |  | 17  (13–21) |  | [23, 28] |
| Percentage of shared injections |  |  |  |  |  |  | 15.4%  (7.1–19.6%) |  |  | 31.7%  (29.9–33.5%) |  | [23, 28] |
| HIV testing rate per year |  |  |  |  |  |  | 8.4%  (7.1–9.6%) |  |  | 31.7%  (29.9–33.5%) |  | [23, 28] |
| Prevalence of sexually transmitted infections |  |  | 20.4%  (16.9–24.0%) |  | 15.1%  (12.0–18.1%) |  | 3.7%  (2.8–4.5%) |  |  | 2.3%  (1.8–2.9%) |  | [23, 28, 43] |

*Street-based female sex worker, among whom sex work largely contributes to their earning, are assumed to be direct FSWs. Entertainment-based FSWs, among whom sex work is a part of their earnings, are assumed to be indirect FSW

**Table E. Healthcare costs of HIV infected people in 2009.**

| **Health utility parameters by populations** | **Average estimate** | **Lower estimate** | **Upper estimate** |
| --- | --- | --- | --- |
| Non-ART cost for PLHIV by CD4 T-cell count |  |  |  |
| >500 cells/µL | $104.6 | $83.1 | $126.1 |
| 350-500 cells/µL | $114.5 | $84.2 | $144.8 |
| 200-350 cells/µL | $127.8 | $84.7 | $171.0 |
| <200 cells/µL | $178.0 | $69.4 | $286.6 |
| Cost of first-line ART | $344.2 | $304.5 | $383.9 |
| Cost of second-line ART | $1,532.8 | $1,351.9 | $1,719.2 |
| Cost of HIV testing per client | $ 7.2 | $5.4 | $9.0 |

Source of data was based on previous work of Duong et al. [63] and personal communication with Duong Thuy Anh.

**Table F. Disability-weights from Global Disease Burden study for cost effectiveness calculations.**

| **Health utility parameters by populations^*^** | **Average estimate** | **Lower estimate** | **Upper estimate** |
| --- | --- | --- | --- |
| Uninfected PWID | 0.0363 | 0.0191 | 0.0588 |
| Untreated HIV-infected population |  |  |  |
| PLHIV with CD4 >500 cells/µL | 0.2000 | 0.1030 | 0.3040 |
| PLHIV with CD4 350-500 cells/µL | 0.2000 | 0.1030 | 0.3040 |
| PLHIV with CD4 200-350 cells/µL | 0.2210 | 0.1460 | 0.3100 |
| PLHIV with CD4 <200 cells/µL | 0.5470 | 0.3820 | 0.7150 |
| Treated HIV-infected population |  |  |  |
| PLHIV on cART with CD4 >500 cells/µL | 0.0363 | 0.0191 | 0.0588 |
| PLHIV on cART with CD4 350-500 cells/µL | 0.0363 | 0.0191 | 0.0588 |
| PLHIV on cART with CD4 200-350 cells/µL | 0.0401 | 0.0271 | 0.0600 |
| PLHIV on cART with CD4 <200 cells/µL | 0.0993 | 0.0708 | 0.1384 |

*Estimates were based on previous work of Salomon et al. [64] and Stouthard et al. [65].

1. Counterfactual Scenarios in the Absence of HIV Interventions

Using a generalized 4-parameter logistic function, for each at-risk populations and general population we calibrated the behavioural parameters from 2000 to 2015 affected by prevention programmes targeting that population in a counterfactual scenario to determine the cost-effectiveness of HIV programme funding in Vietnam. Five HIV prevention programs were evaluated and details of selected key behaviours that are more likely influenced by an intervention are summarized in Table G. These scenarios were based on the assumed effect of the removal of specific programmes. The initial values are assumed parameters at the programme initiation and the saturate/stable values of scenario at 2015 are assumed to be at the similar levels as the staring year of each implementations or locations in the absence of the intervention programmes. All counterfactual scenarios are presented in Fig. 2 in the main text.

**Table G. Selected behaviours affected by HIV prevention programmes.**

| **HIV prevention programme** | **Targeted behaviour** |
| --- | --- |
| 1. Needle-syringe exchange program (NSP) | Percentage of PWID who report sharing injections in the past month |
| 1. Methadone maintenance therapy (MMT) | Percentage of PWID receiving MMT |
| 1. 100% condom use for both direct and indirect FSWs | Percentage of condom use in the last sex acts with clients in both direct and indirect FSWs  Percentage of condom use in the last sex acts with non-commercial partners in both direct and indirect FSWs |
| 1. 100% condom use and lubricant for MSM | Percentage of condom use in the last sex acts with non-commercial male partners in MSM  Percentage of condom use in the last sex acts with commercial male partners in MSM |
| 1. Antiretroviral therapy | Number of people receiving treatment for HIV |

1. Calibration of model to the HIV epidemic in Vietnam

**Fig G. Calibrated input parameters by Optima.**


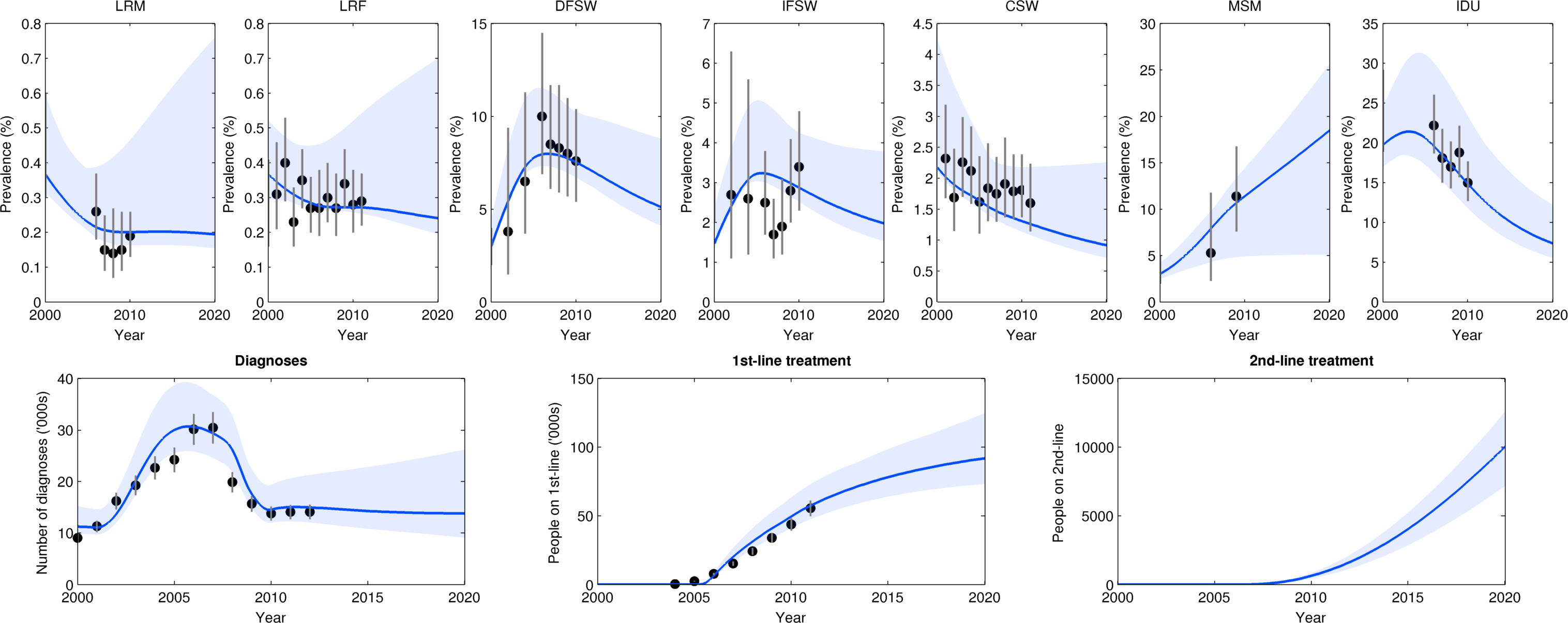


*Note: In each figure, blue solid curve represents best-fitting trend, light blue band represents 95% uncertainty bounds of 40 calibrations closely fitted to observed data. Black dots with solid vertical lines represent observed epidemiological and case-reporting data.*

1. Impact of optimal allocation on HIV prevalence, 2013-2020

**Fig H. Projected prevalence levels are shown by population group.**

1. **Current allocation of HIV prevention funding to programs versus the optimal budget allocation to reduce HIV incidence**

**
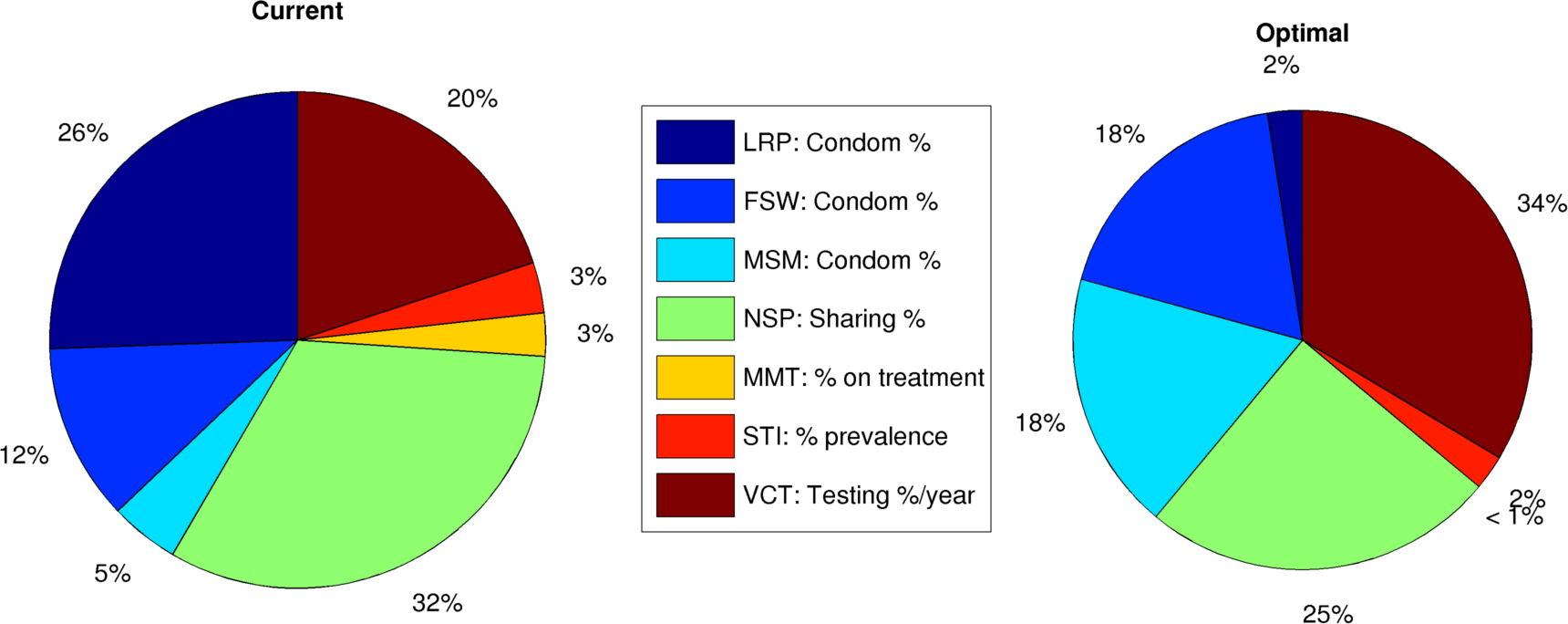
**

1. **National HIV prevalence in 2000-2020**


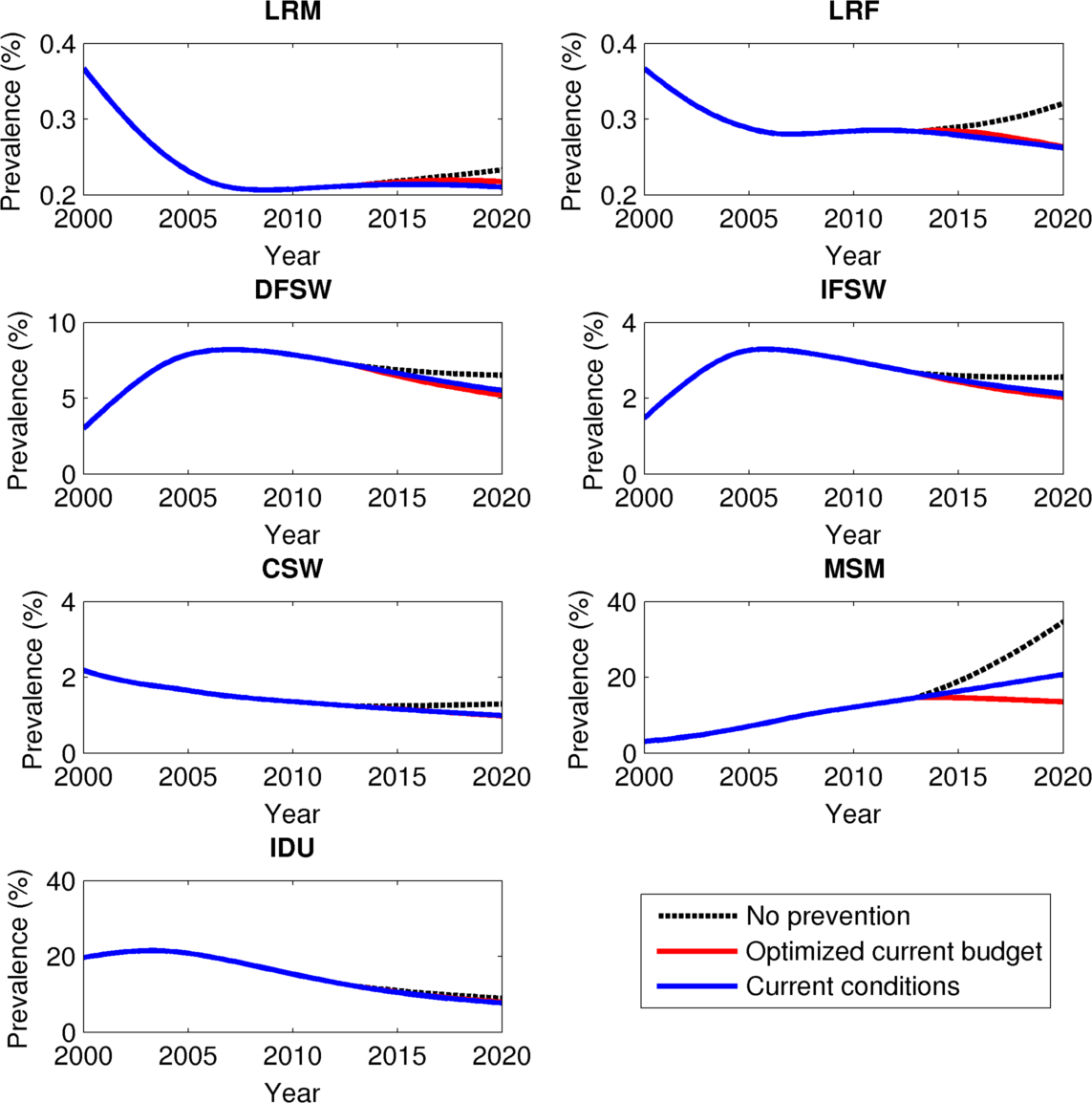


**REFERENCES**

1. Vietnam Administration for HIV/AIDS Control, The World Bank, Wales UoNS. Evaluation of the epidemiological impact of harm reduction programs on HIV in Vietnam. 2001. Available from: <http://kirby.unsw.edu.au/publications/evaluation-epidemiological-impact-harm-reduction-programs-hiv-vietnam-main-report> [cited 2014 September 22].47-9.

2. Nguyen N, Nguyen H, Trinh H, Mills S, Detels R. Clients of female sex workers as a bridging population in Vietnam. AIDS Behav. 2009;13(5):881-91.

3. Nguyen TH, Le TG, Phan NB, Wolffers I. The social context of HIV risk behaviour by drug injectors in Ho Chi Minh City, Vietnam. AIDS Care. 2000;12(4):483-95.

4. Hien N, Giang L, Binh P, Deville W, van Ameijden E, Wolffers I. Risk factors of HIV infection and needle sharing among injecting drug users in Ho Chi Minh City, Vietnam. J Subst Abuse. 2001;13(1-2):45 - 58.

5. Nguyen TA, Hoang LT, Pham VQ, Detels R. Risk factors for HIV-1 seropositivity in drug users under 30 years old in Haiphong, Vietnam. Addiction. 2001;96(3):405-13.

6. Tung ND, Tuan N, Hoang TV, Hien NT, Thang BD, Kane TT, et al. HIV/AIDS behavioral surveillance survey Vietnam, 2000. Hanoi, Vietnam: Ministry of Health, 2001. Available from: <http://www.fhi360.org/en/hivaids/pub/survreports/bssvietnam2000.htm> [cited 2012 July 25].

7. Thao LTL, Lindan CP, Brickley DB, Giang LT. Changes in high-risk behaviors over time among young drug users in South Vietnam: a three-province study. AIDS Behav. 2006;10(Suppl 7):S47-S56.

8. Tuan NA, Fylkesnes K, Thang BD, Hien NT, Long NT, Van Kinh N, et al. Human immunodeficiency virus (HIV) infection patterns and risk behaviours in different population groups and provinces in Viet Nam. Bull World Health Organ. 2007;85:35-41.

9. Tran TM, Nguyen H, Yatsuya H, Hamajima N, Nishimura A, Ito K. HIV prevalence and factors associated with HIV infection among male injection drug users under 30: a cross-sectional study in Long An, Vietnam. BMC Public Health. 2006;6(1):248.

10. Des Jarlais DC, Kling R, Hammett TM, Ngu D, Liu W, Chen Y, et al. Reducing HIV infection among new injecting drug users in the China–Vietnam Cross Border Project. AIDS. 2007;21:S109-S14.

11. Hammett TM, Johnston P, Kling R, Liu W, Ngu D, Tung ND, et al. Correlates of HIV status among injection drug users in a border region of southern China and northern Vietnam. J Acquir Immune Defic Syndr. 2005;38(2):228-35.

12. Bergenstrom A, Go V, Nam LV, Thuy BT, Celentano DD, Frangakis C, et al. Return to post-test counselling by out-of-treatment injecting drug users participating in a cross-sectional survey in north Vietnam. AIDS Care. 2007;19(7):935-9.

13. Quan VM, Go VF, Nam LV, Bergenstrom A, Thuoc NP, Zenilman J, et al. Risks for HIV, HBV, and HCV infections among male injection drug users in northern Vietnam: a case–control study. AIDS Care. 2008;21(1):7-16.

14. Go VF, Frangakis C, Van Nam L, Bergenstrom A, Sripaipan T, Zenilman JM, et al. High HIV sexual risk behaviors and sexually transmitted disease prevalence among injection drug users in Northern Vietnam: implications for a generalized HIV epidemic. J Acquir Immune Defic Syndr. 2006;42(1):108-15.

15. Schumacher CM, Go VF, Van Nam L, Latkin CA, Bergenstrom A, Celentano DD, et al. Social injecting and other correlates of high-risk sexual activity among injecting drug users in northern Vietnam. Int J Drug Policy. 2009;20(4):352-6.

16. Pasteur Institute of Ho Chi Minh City. Evaluating a HIV prevention program for injecting drug users in Soctrang, Vietnam [ongoing collaboration]. 2006.

17. Ministry of Health. Final project report: Community action for preventing HIV/AIDS" in five provinces (Dien Bien, Quang Tri, An Giang, Kien Giang and Dong Thap) in Vietnam. Hanoi, Vietnam, 2006: Ministry of Health.

18. Quan VM, Minh NL, Ha TV, Ngoc NP, Vu PT, Celentano DD, et al. Mortality and HIV transmission among male Vietnamese injection drug users. Addiction. 2011;106(3):583-9.

19. Clatts MC, Giang LM, Goldsamt LA, Yi H. Male sex work and HIV risk among young heroin users in Hanoi, Vietnam. Sex Health. 2007;4(4):261-7.

20. Clatts M, Goldsamt L, Minh Giang L, Colón-López V. Accelerated transition to injection among male heroin initiates in Hanoi, Vietnam: implications for early harm reduction interventions. Journal of community health. 2011;36(6):999-1003.

21. Clatts M, Giang LM, Goldsamt L, Colón-López V. Nonmedical use of promethazine hydrochloride among heroin injectors in vietnam: unrecognized risks and unintended consequences. Subst Use Misuse. 2010;45(4):515-27.

22. Clatts MC, Giang LM, Goldsamt LA, Yi H. Novel heroin injection practices. implications for transmission of HIV and other bloodborne pathogens. Am J Prev Med. 2007;32(Suppl 6):S226-S33.

23. Ministry of Health. Results from the HIV/STI integrated biological and behavioral surveillance (IBBS) in Vietnam, 2005-2006. Ha Noi, Vietnam: Ministry of Health; 2006. Available at: <http://www.inthealth.ku.dk/reach/resources/surveillance.pdf/>. Accessed 15 April 2014.

24. Pasteur Institute of Ho Chi Minh City. HIV prevalence and risk behaviors among injecting drug users in Vinh Long province, Vietnam [ongoing collaboration]. 2007.

25. Pasteur Institute of Ho Chi Minh City. HIV prevalence and risk behaviors among injecting drug users in Ba Ria Vung Tau province, Vietnam [ongoing collaboration]. 2009.

26. Pasteur Institute of Ho Chi Minh City. HIV prevalence and risk behaviors among injecting drug users in Soc Trang province, Vietnam [ongoing collaboration]. 2009.

27. United Nations Office on Drugs and Crime (UNODC). HIV and risk behaviors among injecting drug users in Lang Son, Vietnam. 2009.

28. Ministry of Health. Results from the HIV/STI intergrated biological and behavioral surveillance (IBBS) in Vietnam round II, 2009. Hanoi, Vietnam: Ministry of Health; 2011. Available at: <http://aidsdatahub.org/dmdocuments/Vietnam_IBBS_Round_II_2009.pdf>. Accessed 22 September 2011.

29. Giang LT, Son NT, Thao LTL, Vu L, Hudes ES, Lindan C. Evaluation of STD/HIV prevention needs of low- and middle-income female sex workers in Ho Chi Minh City, Vietnam. AIDS Behav. 2000;4(1):83-91.

30. Nguyen AT, Nguyen TH, Pham KC, Le TG, Bui DT, Hoang TL, et al. Intravenous drug use among street-based sex workers: a high-risk behavior for HIV transmission. Sex Transm Dis. 2004;31(1):15-9.

31. Truong TM, Do TN, West GR, Durant TM, Jenkins RA, Pham TH, et al. Sex workers in Vietnam: how many, how risky? AIDS Educ Prev. 2004;16(5):389-404.

32. Ministry of Health. Baseline survey report: Community action for preventing HIV/AIDS in Lai Chau, Quang Tri, Dong Thap, An Giang, and Kien Giang. Hanoi, Vietnam, 2002. Available from: <http://www.inthealth.ku.dk/reach/resources/baselinesurvey_vn.pdf/>. Accessed 20 August 2012.

33. Le MN, D'Onofrio CN, Rogers JD. HIV risk behaviors among three classes of female sex workers in Vietnam. J Sex Res. 2010;47(1):38-48.

34. Grayman JH, Nhan DT, Huong PT, Jenkins RA, Carey JW, West GR, et al. Factors associated with HIV testing, condom use, and sexually transmitted infections among female sex workers in Nha Trang, Vietnam. AIDS Behav. 2005;9(1):41-51.

35. Tran TN, Detels R, Long HT, Phung LV, Lan HP. HIV infection and risk characteristics among female sex workers in Hanoi, Vietnam. J Acquir Immune Defic Syndr. 2005;39(5):581-6.

36. Tran TN, Detels R, Long HT, Lan HP. Drug use among female sex workers in Hanoi, Vietnam. Addiction. 2005;100(5):619-25.

37. Tran TN, Detels R, Lan HP. Condom use and its correlates among female sex workers in Hanoi, Vietnam. AIDS Behav. 2006;10(2):159-67.

38. Nguyen T, Khuu N, Truong P, Nguyen A, Truong L, Detels R. Correlation between HIV and sexual behavior, drug use, trichomoniasis and candidiasis among female sex workers in a Mekong Delta province of Vietnam. AIDS Behav. 2009;13(5):873-80.

39. Nguyen TV, Thi Le TT, Nguyen AP, Cao V, Van Khuu N, Tham DC, et al. Sexually transmitted infections and risk factors for gonorrhea and chlamydia in female sex workers in Soc Trang, Vietnam. Sex Transm Dis. 2008;35(11):935-40.

40. Thuong NV, Nhung VT, Nghia KV, Tram LT, O'Farrell N. HIV in female sex workers in five border provinces of Vietnam. Sex Transm Infect. 2005;81(6):477-9.

41. Thuong NV, Long NT, Hung ND, Truc LTT, Nhung VTT, Van CTB, et al. Sexually transmitted infections in female sex workers in five border provinces of Vietnam. Sex Transm Dis. 2005;32(9):550-6.

42. O'Farrell N, Vu Thuong N, Van Nghia K, Thu Tram L, Thanh Long N. HSV-2 antibodies in female sex workers in Vietnam. Int J STD AIDS. 2006;17(11):755-8.

43. Vu Thuong N, Van Nghia K, Phuc Hau T, Thanh Long N, Thi Bao Van C, Hoang Duc B, et al. Impact of a community sexually transmitted infection/HIV intervention project on female sex workers in five border provinces of Vietnam. Sex Transm Infect. 2007;83(5):376-82.

44. Johnston LG, Sabin K, Hien MT, Huong PT. Assessment of respondent driven sampling for recruiting female sex workers in two Vietnamese cities: reaching the unseen sex worker. Urban Health. 2006;83(7 SUPPL.):i16-i28.

45. Nemoto T, Iwamoto M, Colby D, Witt S, Pishori A, Le MN, et al. HIV–related risk behaviors among female sex workers in Ho Chi Minh City, Vietnam. AIDS Educ Prev. 2008;20(5):435-53.

46. Colby DJ. HIV knowledge and risk factors among men who have sex with men in Ho Chi Minh City, Vietnam. J Acquir Immune Defic Syndr. 2003;32(1):80-5.

47. Nguyen T, Nguyen H, Le G, Detels R. Prevalence and risk factors associated with HIV infection among men having sex with men in Ho Chi Minh City, Vietnam. AIDS Behav. 2008;12:476 - 82.

48. Colby DJ, Minh TT, Toan TT. Down on the farm: homosexual behaviour, HIV risk and HIV prevalence in rural communities in Khanh Hoa province, Vietnam. Sex Transm Infect. 2008;84(6):439-43.

49. Pham QD, Nguyen TV, Hoang CQ, Cao V, Khuu NV, Phan HT, et al. Prevalence of HIV/STIs and associated factors among men who have sex with men in An Giang, Vietnam. Sex Transm Dis. 2012;39(10):799-806.

50. Hammett TM, Van NTH, Kling R, Binh KT, Oanh KTH. Female sexual partners of injection drug users in Vietnam: an at-risk population in urgent need of HIV prevention services. AIDS Care. 2010;22(12):1466-72.

51. Hammett TM, Kling R, Van Ha NT, Son DH, Binh KT, Oanh KTH. HIV prevention interventions for female sexual partners of injection drug users in Hanoi, Vietnam: 24-month evaluation results. AIDS Behav. 2012;16(5):1164-72.

52. Ministry of Health. HIV sentinel surveillance reports in 1990-2011 [on going collaboration]. Hanoi, Vietnam: Vietnam Authority of HIV/AIDS Control; 2012.

53. The Joint United Nations Programme on HIV/AIDS (UNAIDS). Vietnam national AIDS spending assessment, 2008-2010. Hanoi, Vietnam: UNAIDS; 2012. Available at: <http://www.aidsdatahub.org/vietnam-national-aids-spending-assessment-2008-2010-unaids-2012>. Accessed 25 July 2012. 2012.

54. Government of Socialist Republic of Viet Nam. Declaration of commitment on HIV and AIDS adopted at the 26th United Nations General Assembly Special Session in June 2001 (UNGASS): Reporting periode: January 2008 - December 2009. Available from: <http://www.unaids.org/en/dataanalysis/knowyourresponse/countryprogressreports/2010countries/vietnam_2010_country_progress_report_en.pdf> [cited 2013 August 12]. 2012.

55. Kerr CCA. Optima paper [Under review]. 2014.

56. General Statistic Office (GSO). Monthly statistical information on population and employment. Available from: <http://www.gso.gov.vn/default_en.aspx?tabid=467&idmid=3> [cited 2011 Auguts 20]

57. Ministry of Health. Estimated population size of key affected populations in Vietnam. Hanoi, Vietnam, 2012: Ministry of Heath.

58. General Statistic Office, National Institute of Hygiene and Epidemiology, ORC Marco. Vietnam population and AIDS indicator survey 2005. 2006.

59. Pasteur Institute of Ho Chi Minh City. Household survey on HIV/STI and knowledge, attitudes, and risk behaviours among 15-49 aged populations in Dong Nai, Hau Giang, and Vinh Long, Vietnam, 2008 [ongoing collaboration].

60. Tuan NA, Ha NTT, Diep VTB, Thang PH, Long NT, Huong PTT, et al. Household survey in two provinces in Viet Nam estimates HIV prevalence in an urban and a rural population. AIDS Res Hum Retroviruses. 2008;24(8):1017-26.

61. National AIDS Standing Bureau, Family Health International, United States Agency for International Development. HIV/AIDS behavioral surveillance survey Vietnam 2000. Hanoi, Vietnam; 2001. Available at: <http://www.aidsdatahub.org/behavioral-surveillance-survey-vietnam-2000-bss-round-1-results-national-aids-standing-bureau-fhi-usaid-et-al-2000>. Accessed 25 July 2012.

62. Ruan Y, Qian HZ, Li D, Shi W, Li Q, Liang H, et al. Willingness to be circumcised for preventing HIV among chinese men who have sex with men. AIDS Patient Care STDS. 2009;23(5):315-21.

63. Duong AT, Kato M, Bales S, Do NT, Minh Nguyen TT, Thanh Cao TT, et al. Costing analysis of national HIV treatment and care program in Vietnam. J Acquir Immune Defic Syndr. 2014;65(1):e1-e7.

64. Salomon JA, Vos T, Hogan DR, Gagnon M, Naghavi M, Mokdad A, et al. Common values in assessing health outcomes from disease and injury: disability weights measurement study for the Global Burden of Disease Study 2010. Lancet. 2012;380(9859):2129-43.

65. Stouthard MEA, Essink-Bot M-L, Bonsel GJ, on behalf of the DDWG. Disability weights for diseases: a modified protocol and results for a Western European region. Eur J Public Health. 2000;10(1):24-30.

1. This prevalence was derived from a sample of drug users not reporting injecting. [↑](#footnote-ref-1)
2. This prevalence was derived from a sample of drug users not reporting injecting. [↑](#footnote-ref-2)
